# PROTOCOL: The effects of empowerment‐based nutrition interventions on the nutritional status of women of reproductive age in low‐ and middle‐income countries

**DOI:** 10.1002/cl2.1183

**Published:** 2021-07-06

**Authors:** Alison Riddle, Abby Ramage, Cynthia M. Kroeger, Zulfiqar A. Bhutta, Elizabeth Kristjansson, Monica Taljaard, Carol Vlassoff, Sara Wuehler, Becky Skidmore, Alexandria L. Bennett, Anita Rizvi, Vivian Welch, George A. Wells

**Affiliations:** ^1^ School of Epidemiology and Public Health University of Ottawa Ottawa Canada; ^2^ Independent Consultant Cambridge UK; ^3^ Charles Perkins Centre, Faculty of Medicine and Health, School of Pharmacy The University of Sydney Sydney Australia; ^4^ Centre for Global Child Health The Hospital for Sick Children Toronto Canada; ^5^ Faculty of Social Sciences, School of Psychology University of Ottawa Ottawa Canada; ^6^ Ottawa Hospital Research Institute Ottawa Canada; ^7^ School of Epidemiology and Public Health University of Ottawa Ottawa Canada; ^8^ Nutrition International Ottawa Canada; ^9^ Independent Ottawa Canada; ^10^ School of Psychology University of Ottawa Ottawa Canada; ^11^ Methods Centre Bruyère Research Institute Ottawa Canada

## Abstract

The systematic review will answer the follow research questions: (1) What is the effectiveness of different empowerment approaches employed within nutrition interventions on the nutritional status of women of reproductive age in low‐ and middle‐income countries? (2) What implementation and contextual factors contribute to or detract from the effectiveness of these interventions?

## 1 BACKGROUND

### 1.1 Description of the condition

Malnutrition is a pervasive global health problem with consequences for survival, healthy development, and the economic productivity of individuals and societies. Women of reproductive age (15–49 years), especially in low‐ and middle‐income countries (LMICs), carry a disproportionate and inequitable burden of malnutrition due to physiological, sociocultural, and economic mechanisms (2020 Global Nutrition Report, 2020).

More than 153 million (9.7%) adult women are underweight (BMI < 18.5 kg/m^2^) globally (2020 Global Nutrition Report, 2020). The prevalence of underweight among women 15–49 years exceeds 10% in some sub‐Saharan African and South Asian countries (Black et al., 2013). Almost 40% of adult women are currently categorised as overweight (BMI > 25.0 kg/m^2^), and just over 15% are obese (BMI > 30.0 kg/m^2^) (2020 Global Nutrition Report, 2020). The global prevalence of overweight and obesity is rising steadily, with an increasing burden placed on lower wealth and education groups in lower income countries (2020 Global Nutrition Report, 2020; Finucane et al., 2011). Among women, overweight and obesity exceeds 70% in the Americas and the Caribbean, 40% in Africa, and 20% in Asia (Black et al., 2013). Anaemia, often caused by low iron consumption or absorption, affects 42% of pregnant women in LMICs, largely in South Asian and African low‐income countries (Rahman et al., 2016). No country is currently on target to achieve global anaemia reduction targets among women of reproductive age (2020 Global Nutrition Report, 2020).

The majority of studies examining the consequences of women's malnutrition focus on pregnant and lactating women and their offspring. These include increased risks of pre‐eclampsia, gestational diabetes mellitus, maternal mortality, low birth weight, small‐for‐gestational age births, and neonatal mortality (Black et al., 2013, Doku et al., 2020). However, there are significant gaps in evidence for other points along the life course (Fox et al., 2019). While malnutrition among women has serious repercussions for the health and cognitive development of infants and children, the rise in overweight, obesity and noncommunicable diseases points to the need to consider the importance of women's nutrition beyond their reproductive role.

Social inequities, especially related to gender and women's low status relative men, are important drivers of malnutrition among women (2020 Global Nutrition Report, 2020; Heise et al., 2019; Sen & Östlin, 2008). Gender inequities, defined as unfair treatment of a person due to their sex, gender identity or gender expression (World Health Organization, 2011), inhibit women's decision‐making power and autonomy and their control of resources that are linked to improved nutritional outcomes for themselves and their families (Cunningham et al., 2015).

Conversely, empowering women and girls is associated with improved health and nutrition outcomes for women and their children (Pratley, 2016). Women's empowerment is defined as “the expansion in people's ability to make strategic life choices in a context where this ability was previously denied to them” (Kabeer, 1999). The majority of the literature examining the relationship between women's empowerment and nutrition are focused on children's outcomes (Bhagowalia et al., 2012; Carlson et al., 2015; Cunningham et al., 2015; Jones et al., 2019; Santoso et al., 2019; van den Bold et al., 2013; Yaya et al., 2020), but there are a growing number of exceptions (Alaofe et al., 2017; Malapit & Quisumbing, 2015; Pratley, 2016; Sinharoy et al., 2018; Sraboni & Quisumbing, 2018; Yaya et al., 2020). Positive associations have been found between women's empowerment and women's dietary diversity (Alaofe et al., 2017; Malapit & Quisumbing, 2015; Sinharoy et al., 2018; Sraboni et al., 2014), BMI (Alaofe et al., 2017; Taukobong et al., 2016; Yaya et al., 2020) and anaemia (Taukobong et al., 2016). However, the importance of different dimensions of empowerment in regard to these outcomes varies by context (Taukobong et al., 2016).

Reviews assessing intervention effects of nutrition programs that adopt empowerment strategies are fewer still (Brandstetter et al., 2015; Fox et al., 2019; Kumar et al., 2018). It can be challenging to disentangle the effects of women's empowerment interventions from their cointerventions, especially given the wide array of ways in which empowerment is operationalized in nutrition programs. Approaches include women's groups (Kumar et al., 2018), income‐generating or household food security interventions (Fox et al., 2019), and educational interventions (Brandstetter et al., 2015). Varying definitions of what constitutes empowerment further complicates matters (Brandstetter et al., 2015; Fox et al., 2019; Richardson, 2018). There remains a need to elucidate the way in which different empowerment‐based approaches work to affect change in women's nutrition outcomes, and what contributes to their effectiveness (Taukobong et al., 2016).

The objective of this review is to begin to address some of these challenges and evidence gaps by reviewing the effectiveness of “empowerment‐based” interventions to improve women's nutrition in LMICs. We begin by applying a comprehensive definition of what constitutes an “empowerment‐based approach,” and then assess the effectiveness of the different models of operationalization in improving women's nutrition outcomes.

### 1.2 Description of the intervention

The definition of an empowerment‐based approach has been outlined previously by the authors (Riddle et al., 2019). We consider three components to an empowerment‐based approach based on previous works by Alsop and Heinsohn (2005), Kabeer (1999), Malhotra et al. (2002), and others: fostering agency, creating a supportive opportunity structure, and the achievement of desired outcomes.

Agency is defined as “the ability to define one's goals and act upon them” (Kabeer, 1999). Central to this definition is (1) the availability of alternatives from which to choose, (2) the individual is aware of the alternatives available to them, and (3) the individual desires to make a choice (Alsop & Heinsohn, 2005; Kabeer, 1999). Agency is often described in terms of decision‐making power, but it can also reflect an ability to bargain, negotiate, influence, resist, or manipulate (Kabeer, 1999). An intervention that fosters agency will include activities designed to *increase women's motivation and abilities to make informed decisions by providing spaces for self‐reflection and identification of significant life areas* (Shankar et al., 2019). Such interventions are hypothesised to enable women's active and meaningful participation in decision‐making, instil a sense of self‐efficacy, and increase self‐esteem and motivation to make a positive change in pursuit of strategic life goals. Examples of activities that foster agency are life skills training programs, behaviour change counselling programs that apply established theories to promote self‐efficacy, problem solving, and decision‐making, such as Social Cognitive Theory (SCT) (Bandura, 1992) or the Health Belief Model (Rosenstock, 1974), participatory learning and action women's groups (Prost et al., 2013), and other programs that create “safe spaces” for women or equip women to make informed strategic life decisions.

Opportunity structure is defined as “the formal and informal contexts within which actors operate” (Alsop & Heinsohn, 2005). This component includes access to resources (human, financial, social, material, etc.), and is a precondition to the exercising of agency (Kabeer, 1999). Creating a supportive opportunity structure aims to alter the constraining political, economic, sociocultural, interpersonal, and/or legal structures (informal or formal) at the household, community, or broader societal levels, as necessary, to support women to exercise agency (Alsop & Heinsohn, 2005; Malhotra et al., 2002). The type of activities that are undertaken to create a supportive opportunity structure will vary by context, thus we cannot provide an exhaustive list. Instead, we have attempted to categorise activities by type according to the dimension of empowerment they seek to redress.

- *Economic*. Economic activities aim to increase women's access to and control over financial and material resources. These include microcredit programs, cash transfer programs, agriculture programs, homestead or community gardening programs, and savings and loan programs.
- *Sociocultural*. Sociocultural activities aim to redress discriminatory gender norms, customs and practices that restrict women's ability to exercise agency, most often at the household and community level. Examples include programs to keep adolescent girls in school, to improve women's freedom of movement in the community, to delay ages of first marriage and first birth, or to engage community leaders and other key community influencers (leaders, traditional healers, etc.) in supporting women to exercise agency over their nutritional status.
- *Legal*. Legal activities aim to establish laws meant to prevent gender‐based discrimination and protect women's rights. This can include women's rights to education, family planning, employment, inheritance, and laws to prevent child marriage.
- *Political*: Political activities aim to include women in political processes and to support their ability to self‐organise.
- *Intra‐familial*: Intra‐familial activities aim to redress unequal social hierarchy and dynamics within the household. An example is engaging husbands, partners, or mothers‐in‐law in the support of women's agency and autonomy.

Agency and opportunity structure interact to contribute to the achievement of an individual's desired outcomes, as they themselves define them. The absence of activities to either foster agency or opportunity structure is proposed to be suboptimal to the achievement of desired outcomes. Thus, an empowerment‐based nutrition intervention must include activities to foster agency and create a supportive opportunity structure.

This review will examine the effects of an empowerment‐based approach in nutrition interventions intended to improve dietary intake or micronutrient status in women of reproductive age in LMICs. To manage the review's scope, we define eligible nutrition interventions as those that aim to improve dietary intake or micronutrient status. Examples of eligible nutrition interventions are nutrition education or counselling (standalone in combination with other activities), micronutrient supplementation (iron, folic acid, vitamins A, D, etc.), food fortification at point of consumption, and food supplementation.

An example of a primary study that may be included in this review is a cluster randomized controlled trial (RCT) conducted in Nepal of participatory learning action (PLA) women's groups (agency component) for the delivery of nutrition education (nutrition component) paired with either an unconditional food transfer (additional nutrition component) or an unconditional cash transfer (opportunity structure component—economic dimension) (Harris‐Fry et al., 2018). The control group was the provision of the government's usual services. Participants were pregnant women of reproductive age. The study assessed mid‐upper arm circumference (MUAC), food and nutrition intakes, and intra household food allocation.

### 1.3 How the intervention might work

**Figure 1:**
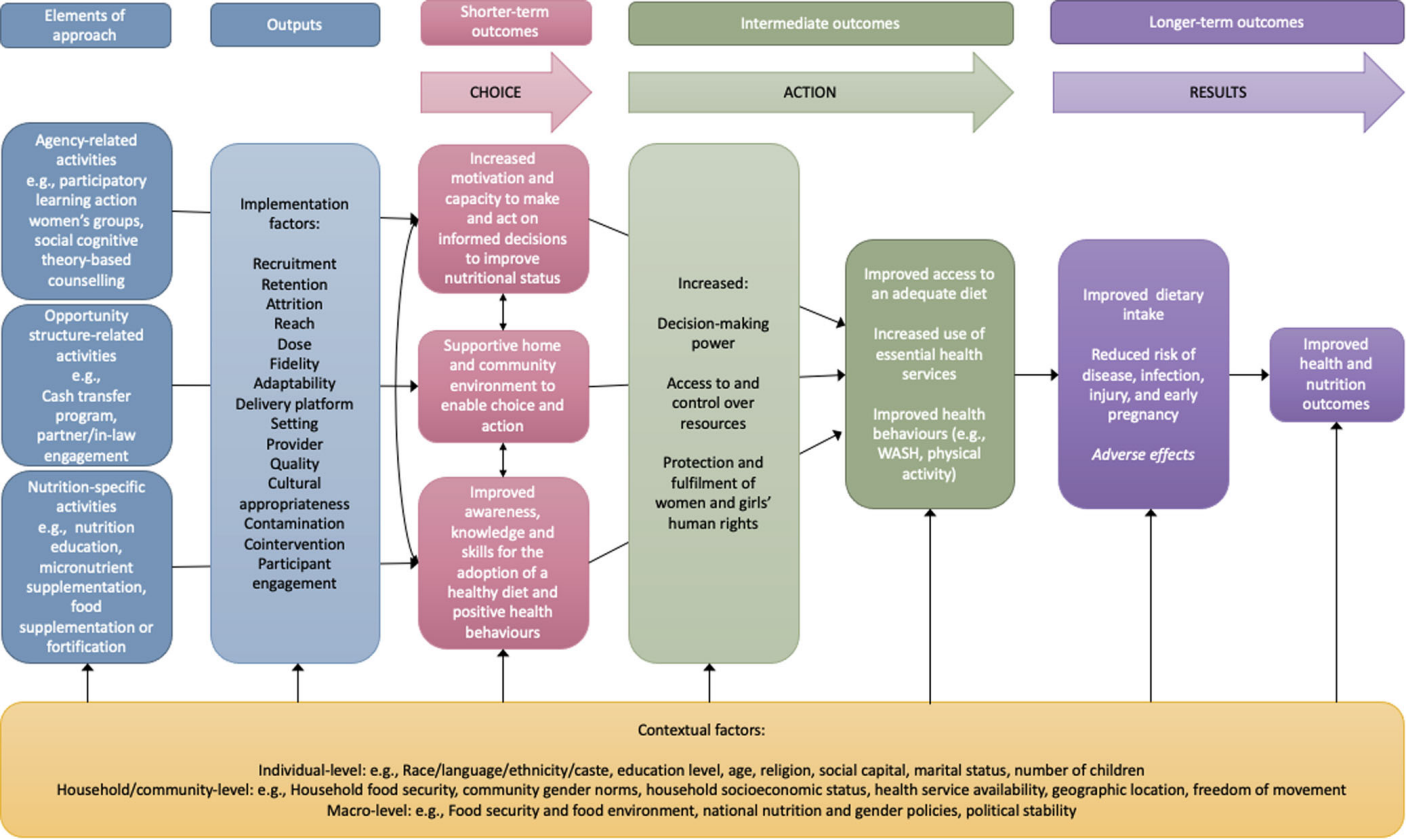
Logic model: Empowerment‐based nutrition interventions to improve women's nutritional status (adapted from Riddle et al., 2019)

Our logic model is based on a previously published model for a companion review of the effects of empowerment‐based nutrition intervention on the nutritional status of adolescent girls in LMICs (Riddle et al., 2019). More details on the model's development and components can be found there. We have updated model to focus on our population of interest (women of reproductive age) (Figure 1). The model depicts the causal pathways from the implementation of an empowerment‐based nutrition intervention to improved nutrition outcomes for women.

The model begins with the implementation of the *three elements* of an empowerment‐based nutrition intervention: nutrition‐related activities, agency‐related activities, and opportunity structure related activities.

The effectiveness of these activities will be influenced by several *implementation factors*: the intervention setting and provider, recruitment and retention strategies, the level of attrition, intervention reach and intensity, fidelity of implementation, adaptation to local context, cultural appropriateness, potential for contamination by other interventions, the provision of cointerventions, and participant engagement (Cargo et al., 2018).

The three elements of an empowerment‐based approach are then hypothesised to lead to *three short‐term outcomes*, respectively:

1. Improved awareness, knowledge and skills for the adoption of a healthy diet and positive health behaviours (nutrition‐related activities).
  For example, the provision of a nutrition education program that includes a cooking demonstration will increase women's knowledge about what constitutes a healthy diet and improve her food preparation skills.
2. Increased motivation and capacity to make and act on informed decisions to improve nutritional status (agency‐related activities).
  Building on the previous example, if the nutrition education program is designed and delivered using social cognitive theory (Bandura, 1992), it will include activities to build women's self‐efficacy and self‐confidence so that they can successfully put what they have learned to use.
3. Supportive home and community environment to enable choice and action (opportunity structure‐related activities).

To complete our example, the program could engage women's marital partners, in‐laws or other key influencers and decision‐makers in the household or community to sensitize them to women's nutritional requirements and to garner their support for women taking action to improve their dietary intake.

At the *intermediate outcome* level, fostering women's agency and creating a supportive opportunity structure is hypothesised to contribute to women's empowerment and lead to improvements in their decision‐making power, access to and control over resources, and the protection and fulfilment of their human rights, for example, their right to access education and health care or their freedom of movement. The dimensions in which these changes occur will depend on the context and the gender inequities that disempower women in that particular context.

Returning to our previous example, the combination of nutrition education, building self‐efficacy through SCT, and sensitizing key influencers is meant to improve women's decision‐making power over what and when she eats, increase her control over household resources to purchase and prepare healthy foods for herself and her family, and shift household norms to protect her right to an adequate diet (e.g., by influencing household heads to shift intra‐household food allocation practices so that women no longer eat last and least).

This, in turn, contributes to redressing the underlying drivers of malnutrition among women by:

1. Improving their access to an adequate diet
2. Increasing their use of essential health services, such as antenatal care
3. Improving the adoption of positive health behaviours, such as handwashing

Potential adverse outcomes of empowerment must also be considered at the intermediate level. For example, women may face increased levels of intimate partner violence in response to their attempts to exert greater autonomy, or they may opt to increase their intake of sugar sweetened beverages.

In the *longer‐term*, these actions are hypothesised to lead to improved dietary intake, a reduced risk of disease, infection, or injury, and ultimately, improved health and nutrition outcomes.

*Contextual factors* are hypothesised to moderate the effects of empowerment‐based nutrition interventions. They can occur at three levels and must be considered when examining the impacts of empowerment‐based nutrition interventions as they can lead to differential effects:

1. *Individual‐level* characteristics that can contribute to disadvantage and health inequities, such as education level, socioeconomic status, and place of residence.
2. *Household/community‐level* characteristics, such as household food insecurity, intra‐household food distribution, and the availability of health services in the community.
3. *Macro‐level* characteristics, including national health, gender, and food policies.

### 1.4 Why it is important to do this review

There are few existing reviews that examine the effects of nutrition interventions for women that employ empowerment strategies. We identified four existing reviews consider women's empowerment and nutrition outcomes among women. However, we did not find any reviews that provided a comprehensive model for what comprises an empowerment‐based approach in the context of women's nutrition programming. In contrast, the goal of our review is to use a comprehensive definition that delineates the different components of an empowerment‐based approach to identify and assess interventions that apply a theoretically informed approach to empowerment in the context of nutrition programming for women of reproductive age.

#### 1.4.1 Summary of existing reviews

Fox et al. (2019) summarised the evidence for nutrition‐specific and nutrition‐sensitive interventions (including empowerment) for women across the life course. They found some evidence of an association between women's empowerment and increased income allocated to food expenditures, improved household food security, and increased dietary diversity. The most common delivery platform was community‐based programs, and interventions largely targeted adult women with children. However, their review did not expand on what constituted an empowerment intervention, nor explore the effects of different empowerment approaches on women's nutrition.

Brandstetter et al. (2015) summarised the ways in which empowerment is operationalized in nutrition interventions in high‐income countries and found five (quasi‐)experimental intervention studies that used empowerment messaging. The ways the messaging was delivered varied considerably, from encouraging participants to articulate their concerns and advocate for healthy nutrition in their communities to women being taught skills to achieve change at the personal and community levels. The studies were not exclusively focused on women's empowerment (Brandstetter et al., 2015).

Ruel and colleagues reviewed the effectiveness of nutrition‐sensitive interventions (agricultural programmes, social safety nets, early child development and schooling), including their impact through women's empowerment (Ruel & Alderman, 2013). Their review did not examine the impact of integrating women's empowerment approaches in nutrition‐specific interventions to improve dietary intake but did find that agriculture and social safety net programs likely contribute to improved nutrition through empowerment.

A review by Kumar et al. (2018) synthesized the evidence surrounding the impacts of women's group programs and women's group membership on maternal and child nutrition in South Asia to inform the development of a conceptual framework depicting the pathways through which women's groups lead to improved nutrition outcomes (Kumar et al., 2018). They identified 36 eligible studies of varying study design that assessed 167 different nutrition outcomes. They found evidence of a positive/significant association for 40 (24%) outcomes, 11 (7%) with a negative/significant association, and 116 (69%) with nonsignificant findings.

#### 1.4.2 Existing protocols

This systematic review is modelled after another Campbell systematic review currently in progress by the same authors (Riddle et al., 2019). The companion review examines empowerment‐based nutrition interventions for a different population of interest—adolescent girls (10–19 years old). The reviews were separated because the nutrition interventions, delivery platforms, and empowerment‐based approaches for women of reproductive age and adolescent girls are different (Bhutta et al., 2013). For example, nutrition interventions for adolescent girls are often school‐based, while common delivery platforms for interventions targeting women of reproductive age are the community and health facilities.

## 2 OBJECTIVES

The systematic review will answer the follow research questions:

1. What is the effectiveness of different empowerment approaches employed within nutrition interventions on the nutritional status of women of reproductive age in low‐ and middle‐income countries?
2. What implementation and contextual factors contribute to or detract from the effectiveness of these interventions?

## 3 METHODS

### 3.1 Criteria for considering studies for this review

#### 3.1.1 Types of studies

We will conform to the Cochrane Collaboration's Effective Practice and Organisation of Care (EPOC) criteria for the selection of studies (EPOC, 2017). The following experimental and quasi‐experimental study designs will be included to answer Research Question 1:

- RCTs
- Cluster‐RCTs (cRCTs)
- Controlled before and after studies (CBAs)
- Interrupted time series (ITS)
- Controlled interrupted time series (cITS)
- Other quasi‐experimental designs with at least one comparison group or appropriate methods to control for selection bias, such as Propensity Score Matching (PSM) on baseline covariates, difference‐in‐differences (DID) analysis, or regression discontinuity design (RDD).

To answer Research Question 2, we will include companion quantitative and qualitative studies/papers that assess the contextual and implementation factors influencing the effectiveness of the studies screened in to the effectiveness assessment. We will include qualitative studies that explore the perspectives of intervention participants, providers, or administrators on the contextual and implementation factors related to intervention effectiveness using focus groups, in‐depth interviews or participant observation. Qualitative studies that do not report a clear methods and results section will be excluded (e.g., opinion pieces and editorials). Eligible quantitative study designs that assess the implementation of included studies are process evaluations, surveys of intervention participants, providers, administrators, and other programme‐related documents such as monitoring and evaluation reports.

Studies that apply a mixed methods design will be included where the qualitative and quantitative study components are reported separately (i.e., in separate manuscripts or reported separately in one manuscript).

#### 3.1.2 Types of participants

The population of interest is women of reproductive age (15–49 years old) in low income, lower‐middle income and upper‐middle income countries, according to the World Bank classification of countries by income at the time data were collected. The income classification is based on the gross national income per capita, adjusted for exchange rate fluctuations and inflation (World Bank, n.d.). Studies undertaken in high‐income countries will be excluded.

For studies that include participants outside the 15–49‐year age range, we will include the study if <10% of the participants are outside this range. Alternatively, if *more* than 10% are outside the 15–49‐year range, we will contact the authors to find out if they can share the data that applies exclusively to our population of interest. If this data is not available, then the study will be excluded.

No restrictions on health status will be applied. Pregnant and non‐pregnant participants will be analysed separately.

#### 3.1.3 Types of interventions

Eligible studies must include all three components of an empowerment‐based nutrition intervention: (1) activities to improve dietary intake or micronutrient status, (2) activities to foster agency, and (3) activities to create a supportive opportunity structure. The examples provided below are not exhaustive as there many be ways that agency and opportunity structure, in particular, may be operationalized that the authors have not accounted for.

Nutrition‐related activities to improve dietary intake may be aimed at addressing undernutrition (e.g., through micronutrient supplementation) or overnutrition (e.g., nutrition education to prevent or treat overweight and obesity). However, these interventions will be analysed separately. Eligible activities to improve dietary intake or micronutrient status include:

- Iron and folic acid supplementation
- Multiple micronutrient supplementation
- Calcium supplementation
- Balanced energy protein supplementation
- Nutrition education and counselling
- Food supplementation
- Food fortification at the point of consumption

Eligible activities to foster agency must *increase women's motivation and abilities to make informed decisions by providing spaces for self‐reflection and identification of important life areas* (Shankar et al., 2019). These include:

- Women's groups, such as PLA groups, or other peer‐support group activities
- Behaviour change counselling for nutrition (individual or group) that promotes motivation, problem‐solving, self‐efficacy, or decision‐making (e.g., SCT, Theory of Planned Behaviour, Health Belief Model)
- Life skills training that promotes leadership and confidence building
- Other activities that create “safe spaces” for self‐reflection, problem solving, and decision‐making

Eligible activities to create a support opportunity structure intend to *alter the constraining political, economic, sociocultural, interpersonal, and/or legal structures (informal or formal) at the household, community, or broader societal levels, as necessary to support women to exercise agency* (Alsop & Heinsohn, 2005; Malhotra et al., 2002). The type of activities will vary by context, but are hypothesised to fall into one of the following dimensions:

- *Economic*. Economic activities aim to increase women's access to and control over financial and material resources. These include microcredit programs, cash transfer programs, agriculture programs, homestead or community gardening programs, and savings and loan programs.
- *Sociocultural*. Sociocultural activities aim to redress discriminatory gender norms, customs and practices that restrict women's ability to exercise agency, most often at the household and community level. Examples include programs to keep adolescent girls in school, to improve women's freedom of movement in the community, to delay ages of first marriage and first birth, or to engage community leaders and other key community influencers (leaders, traditional healers, etc.) in supporting women to exercise agency over their nutritional status.
- *Legal*. Legal activities aim to establish laws meant to prevent gender‐based discrimination and protect women's rights. This can include women's rights to education, family planning, employment, inheritance, and laws to prevent child marriage.
- *Political*: Political activities aim to include women in political processes and to support their ability to self‐organise.
- *Intra‐familial*: Intra‐familial activities aim to redress unequal social hierarchy and dynamics within the household. An example is engaging husbands, partners, or mothers‐in‐law in the support of women's agency and autonomy.

Nutrition‐sensitive interventions, such as water, sanitation, and hygiene (WASH) programs, agriculture programs including community gardens, cash transfer programs, food security programs, and family planning programs will be included if they are part of a complex intervention that includes all three elements of an empowerment‐based nutrition intervention as defined above.

##### Comparison

Eligible comparators are (1) nutrition interventions that do not include an empowerment approach (i.e., no agency‐ or opportunity structure‐related activities) or (2) usual care.

#### 3.1.4 Types of outcome measures

##### Primary outcomes

Primary quantitative outcomes will be measures of nutritional status, including:

- Body mass index (BMI) (kg/m^2^), including dichotomous measures for underweight (BMI < 18.5 kg/m^2^), overweight (BMI > 25 kg/m^2^), and obesity (BMI > 30 kg/m^2^). For adolescents, we will use the following cut‐offs: underweight (−2 *SD* from reference mean), stunting (−2 *SD* from reference mean), overweight (+1 *SD* from reference mean), obesity (+2 *SD* from reference mean)
- Weight (kg)
- Haemoglobin (g/L)
- Anaemia (Hb <110 g/L for pregnant women, and Hb <120 g/L for nonpregnant women)
- MUAC (cm)

##### Secondary outcomes

Secondary quantitative outcomes are:

- Dietary diversity or intake, measured using a validated scale (e.g., Dietary Diversity Score), including measures of dietary intake related to individual food groups and intake of unhealthy food groups, such as sugar‐sweetened beverages
- Nutrition‐related knowledge and attitudes, measured using a validated scale
- Validated measures of self‐efficacy, autonomy, decision‐making, for example, the Relative Autonomy Index
- Measures related to unintended consequences of an empowerment‐based approach, such as gender‐based violence/intimate partner violence

Eligible qualitative outcomes are the views, experiences, and opinions of programme implementers and programme participants regarding the feasibility, appropriateness, and meaningfulness of the empowerment‐related approaches integrated in the nutrition intervention.

###### Duration of follow‐up

We will include studies of any follow‐up duration and will conduct sensitivity analyses by length of follow‐up to test the sustainability of treatment effect. Outcomes assessed immediately following the intervention and post‐intervention (e.g., 1 year after the intervention has ended) will be included and reported separately.

###### Types of settings

Interventions delivered at home, in the community, in the workplace, or in health facilities will be eligible for inclusion.

###### Language

No language restrictions will be applied.

###### Publication date

No publication date restrictions will be applied.

### 3.2 Search methods for identification of studies

The methods described below are the same as those published in our companion review (Riddle et al., 2019).

#### 3.2.1 Electronic searches

A comprehensive search strategy will be developed with the assistance of an information specialist (Appendix A). A second senior information specialist will peer review the search strategy prior to execution using the PRESS Checklist.

We will use a combination of controlled vocabulary (e.g., “Power (Psychology),” “Women's Rights,” “Nutritional Status”) and key words (e.g., empower, female status, diet) for the concepts in all searches. We will apply the Cochrane filter for low‐ and middle‐income countries. Vocabulary and syntax will be adjusted as necessary across the databases. We will remove animal‐only and opinion pieces from the results.

Using the OVID platform, we will search Ovid MEDLINE®, including Epub Ahead of Print and In‐Process & Other Non‐Indexed Citations, Embase Classic + Embase, PsycINFO, and the following EBM Reviews databases: Cochrane Central Register of Controlled Trials, Cochrane Database of Systematic Reviews, Database of Abstracts of Reviews of Effects, Health Technology Assessment, and the NHS Economic Evaluation Database. We will also search CINAHL (Ebsco platform), Web of Science, and Popline.

We will document the search process in enough detail to ensure that it can be reported correctly in the review/update, including reporting the month and year the search began and ended.

#### 3.2.2 Searching other resources

To identify potentially relevant unpublished materials, we will consult the following online platforms and websites:

- World Health Organisation Library and the WHO Global Index Medicus
- Eldis.org
- Epistemonikos
- 3ie Impact and Systematic Review repositories
- E‐Library of Evidence for Nutrition Actions (eLENA)
- UNICEF
- World Food Programme
- UNFPA
- International Food Policy Research Institute (IFPRI)
- Global Alliance for Improved Nutrition (GAIN)
- Nutrition International
- USAID/FANTA Project/SPRING Project
- International Center for Research on Women (ICRW)
- UN Women
- U.K. Department for International Development (DFID)
- Bill and Melinda Gates Foundation
- Plan International
- CARE
- Save the Children
- World Vision
- Emergency Nutrition Network
- Food and Agriculture Organisation
- Alive and Thrive
- Nutrition Exchange/Field Exchange

Citation and reference lists:

The citation and reference lists of included studies and other reviews will be searched. “Related articles” features of searched databases will be used, where applicable. We will conduct forward citation tracking using Scopus.

##### Contacting experts

We will contact authors of included studies to ask for suggested studies.

### 3.3 Data collection and analysis

#### 3.3.1 Description of methods used in primary research

The following study is an example of the expected eligible primary research methods:

Olney et al. (2016) used a cRCT in Burkina Faso to test an intervention to improve mothers' nutritional outcomes and empowerment through an enhanced homestead food production intervention (opportunity structure component—economic dimension—increase in asset ownership) alongside a nutrition education intervention that applied the Essential Nutrition Actions framework (nutrition education component). Participating women were invited to participate in mother's groups where a behaviour change communication strategy was employed to empower women (agency‐related component). The authors used DID estimates to assess the impacts on mothers’ dietary intake, diversity, body mass index, and prevalence of underweight (BMI < 18 kg/m^2^).

#### 3.3.2 Criteria for determination of independent findings

We will employ the same criteria for the determination of independent findings as in Riddle et al. (2019).

Where studies report different outcomes, these will be pooled in separate meta‐analyses. If there are several publications reporting on the same study, we will use effect sizes from the most recent publication. In cases where several studies use the same data set or multiple outcomes are reported within the same study, we will select the study that provides the lowest risk of bias in attributing impact. Where studies include multiple outcome measures to assess related outcome constructs, we will select the outcome that appears to most accurately reflect the outcome construct of interest. For studies in which multiple effects over time are reported, a meta‐regression incorporating time will be conducted. For studies having multiple treatments with only one control group, where the treatments might represent separate treatment constructs, we will calculate the effect size for each pair of treatment versus control separately.

#### 3.3.3 Selection of studies

Study selection will be conducted in duplicate by two independent reviewers using the Covidence platform (www.covidence.org). Titles and abstracts resulting from the search strategy will be independently screened by two reviewers in the first phase, followed by independent full‐text review of eligible studies, also in duplicate. Any discrepancies between the independent reviewers will be resolved by consensus, and in cases of disagreement, a third author will be consulted. A PRISMA study selection flow chart (Moher et al., 2009) will be prepared, and a list of excluded studies will be compiled detailing the reason for each study's exclusion.

We recognise that some studies may apply an empowerment‐based approach without calling it such, and may omit a sufficient description of the intervention in the abstract to allow us to determine study eligibility. Consequently, at the title and abstract screening phase, we will not exclude studies that do not appear to meet our eligibility criteria for agency‐related and opportunity structure‐related activities. These criteria will be assessed at the full‐text stage.

In instances where articles do not provide a sufficient description of the intervention to determine its eligibility, we will look for companion articles describing the intervention or we will contact the authors for additional information. Where we cannot obtain additional information on the intervention, the studies will be categorised as “awaiting assessment” until more information is available.

Reported outcome measures will not be used to determine study eligibility. If studies do not report on a primary outcome of interest, we will contact authors to determine if the data were collected but not reported. If we cannot make this determination or if the primary authors report that the data were not collected, the study will be excluded.

#### 3.3.4 Data extraction and management

Data extraction will be conducted in duplicate by two independent reviewers using an Excel spreadsheet. Both reviewers will use a prepiloted data extraction form. Discrepancies between the two extractors will be resolved through discussion or by consultation with a third reviewer. See Appendix B for draft codebooks that will guide data extraction.

The following information on intervention design will be extracted:

- Description of the nutrition component (e.g., nutrition education, micronutrient supplementation)
- Description of the agency component (e.g., participatory learning and action women's group)
- Description of the opportunity structure component (e.g., cash transfer program, homestead food production)
- Description of control/comparison group(s)
- Any theoretical models used to inform intervention design (e.g., SCT)
- Authors’ definition of empowerment and/or rationale for incorporating agency‐ and opportunity structure‐related activities
- Geography, for example, country, region, city, rural/urban/remote
- Intervention setting, for example, community, home‐based, health facility
- Study duration
- Description of the study population (e.g., age, marital status, health status ‐ pregnant/not pregnant), including inclusion and exclusion criteria
- Study design (e.g., cRCT, CBA)

Implementation and contextual factors to be extracted (if/as described by study authors) (Montgomery et al., 2013):

- The intervention administrator, for example, foreign government, national or local government, nongovernmental organisation, community‐based organisation, and so forth
- The intervention provider, for example, community health worker, health facility staff, peers, and so forth
- Descriptions of any training given to intervention providers before and during the intervention
- Description of any prior needs assessment to inform intervention design
- Participant recruitment procedures
- Participant attrition rate and reasons for attrition
- Activities undertaken to design the intervention in a culturally sensitive manner
- Intervention reach (the degree to which participants are present and participate)
- Intervention dose (frequency, intensity and duration of intervention delivery to participants)
- Intervention integrity/fidelity (degree to which the intervention was delivered according to original design)
- Intervention adaptation (adaptation during implementation to respond to changing circumstances)
- Contamination (unintentional delivery of intervention to comparison group or failure to provide intervention to intervention group)
- Cointervention (unintentional delivery of another intervention to study population)
- Participant engagement (active participation and receptivity to the intervention)
- Intervention quality (quality of intervention materials and resources)
- Any contextual factors that may shape implementation effectiveness (e.g., community level of food insecurity)

For quantitative outcomes, we will extract the following:

For dichotomous outcomes, we will extract the total number of participants in the treatment group and the total number experiencing the event to allow the calculation of odds ratios and relative risks (or data necessary for their calculation).

For normally distributed, continuous outcomes, we will extract means, standard deviations (or data necessary for their estimation) and the number of participants in each treatment group.

For skewed continuous data, we will extract medians, ranges, and *p* values.

Outcomes that were measured at different time points will be recorded separately.

We will extract data on socioeconomic status, education level, race/ethnicity/caste, place of residence (urban, rural, slum, remote), and other potential effect moderators for subgroup analyses based on the PROGRESS‐Plus framework (O'Neill et al., 2014).

Quantitative data will be entered into Review Manager Web version 5 (RevMan5) (Cochrane Collaboration, 2019) and checked for accuracy.

For qualitative studies, we will extract the views, experiences, and opinions of intervention participants, implementers and administrators on factors influencing the success or failure of interventions. Emphasis will be placed on ascertaining the feasibility, appropriateness, and meaningfulness of the agency and opportunity structure components of the intervention.

#### 3.3.5 Assessment of risk of bias in included studies

We will use the risk of bias tool developed by the Secretariat of the International Development Coordinating Group (IDCG), as done by Baird et al. (2013). This tool has been developed to assess the risk of bias for a range of experimental and quasi‐experimental studies. The tool assesses risk of bias in the following categories:

- Selection bias and confounding
- Spill‐overs/crossovers/contamination
- Outcome reporting
- Analysis reporting
- Other risks of bias, including unit of analysis errors, detection bias and placebo effects, motivation and courtesy biases, coherence of results, and others.

Judgements made on risk of bias in quantitative studies will be supported by specific information extracted from the study being assessed.

We will assess the quality of qualitative studies using the Critical Appraisal Skills Programme (CASP) qualitative appraisal research tool (Critical Appraisal Skills Programme, 2013).

#### 3.3.6 Measures of treatment effect

Binary outcomes (e.g., anaemia status) will be analysed using risk ratios (±95% confidence interval [CI]). Continuous outcomes (e.g., height, weight) will be analysed using mean differences (±95% CI) and standardised mean differences when different units are used (e.g., measures of dietary diversity or empowerment indicators).

#### 3.3.7 Unit of analysis issues

Unit of analyses will be investigated to ensure estimates are properly adjusted for clustering. Where analyses are not adjusted for clustering, we will look for intra‐cluster coefficient coefficients (ICC) estimates for similar studies in order to approximate a correct analysis (Higgins et al., 2019). Where an appropriate ICC cannot be found, we will exclude the study from our main analysis and conduct a sensitivity analysis using an estimated, plausible ICC.

#### 3.3.8 Dealing with missing data

An intention‐to‐treat analysis will be conducted. We will document how authors treated missing data, and the effect of missing data on the overall results will be assessed through sensitivity analysis. If studies conducted a per protocol analysis, we will ask authors if they also conducted an ITT analysis and are willing to share the data. If not, we will conduct a per protocol analysis.

#### 3.3.9 Assessment of heterogeneity

We will assess heterogeneity among studies by first examining the heterogeneity at face‐value in terms of the studies’ populations, interventions, and outcomes. We will examine *τ* ^2^ and *I* ^2^ and conduct a visual inspection of forest plots to assess heterogeneity, taking into consideration that these measures can be uncertain with few studies.

#### 3.3.10 Assessment of reporting biases

If more than ten studies meet our eligibility criteria, we will assess the presence of publication bias using a visual inspection of funnel plots.

#### 3.3.11 Data synthesis

Similar to our companion review (Riddle et al., 2019), this review will apply a segregated mixed methods research synthesis design (MMRS) (Heyvaert et al., 2017). The approach applies a mixed‐methods approach to the synthesis of quantitative, qualitative and mixed‐methods primary study evidence. Quantitative and qualitative studies are segregated at screening and analysed and synthesized separately. The review's discussion and conclusion draw on both syntheses. Statistical support will be provided by a statistician, and meta‐analyses will be conducted using RevMan5 software.

Quantitative data will be synthesized using meta‐analysis. We will use a random‐effects model to produce an overall summary estimate for each outcome.

We expect a high level of clinical diversity given the wide range of activities that can fall within each component of our intervention (e.g., the nutrition‐related component may be the provision of nutrition education alone or in tandem with micronutrient supplementation). In addition, an objective of our review is to examine how different empowerment approaches may improve women's nutritional status. As such, we will subgroup interventions for analysis as follows:

1. We will separately synthesize interventions that provide nutrition education only from interventions that provide direct dietary or micronutrient support (e.g., provision of micronutrient or food supplementation) because we expect the latter to have more direct effects on nutritional status (e.g., the provision of iron supplementation will be have a greater direct impact on Hb that education only).
2. We will separately synthesize interventions that provide economic support (e.g., cash‐based transfer or livelihood support as an opportunity structure‐related activity) from other forms of opportunity structure‐related support (e.g., such as male partner engagement) because economic empowerment is expected to have a more direct impact on nutritional status (e.g., by providing the program participant with direct access to resources to improve dietary intake).
3. We will separately synthesize studies that provide individual‐focused agency‐related activities (e.g., one‐on‐one counselling), group‐focused activities (e.g., women's groups), and studies that provide both.

If meta‐analysis is not considered optimal (e.g., due to incomplete outcome reporting, limited evidence, or different effect measures), we will summarise effect estimates and present them in tabular format to improve transparency for readers by linking the effects to the studies (McKenzie & Brennan, 2021). We will report our alterative synthesis methods according to the Synthesis without Meta‐Analysis (SWiM) in Systematic Reviews Reporting Guideline (Campbell et al., 2020). In some cases, meta‐analysis and synthesis using another method may be employed depending on the information available for each outcome.

The accuracy of numeric data will be checked by comparing the magnitude and direction of effects reported by studies and how they are presented in the review. A statistically nonsignificant *p* value will be interpreted as a finding of uncertainty unless CIs are sufficiently narrow to rule out an important magnitude of effect.

We will combine experimental and quasi‐experimental designs for analysis and conduct a sensitivity analysis by study design. We will use David Wilson's effect size calculator for quasi‐experimental study outcomes to allow for combining of experimental and quasi‐experimental study outcomes for meta‐analysis (Lipsey, 2001).

Primary and secondary outcome data will be extracted and analysed separately. In the random effects meta‐analysis, Mantel‐Haenszel (M‐H) methods will be used for binary outcomes, and the Inverse‐Variance (I‐V) method will be used for continuous outcomes. Where studies use different metrics for the same outcome, e.g., anaemia status (binary) vs haemoglobin (continuous), we will convert to the same metric using Borenstein's conversion formulae (Borenstein et al., 2009) and synthesize.

##### Treatment of qualitative research

We will use the “best fit” framework synthesis method to synthesize data (Carroll et al., 2013; Harden et al., 2018) according to the review's logic model (Figure 1). We will code qualitative data from the review's included studies against the logic model at each level: approach elements, outputs, short‐term outcomes, intermediate term outcomes, longer‐term outcomes, and contextual factors.

Inductive, thematic analysis techniques will be used to synthesize data that do not align to the existing themes in the logic model. Emphasis will be placed on understanding the role that promoting women's empowerment in the nutrition intervention had on intervention effectiveness, with a focus on aspects regarding the feasibility, appropriateness and meaningfulness of the empowerment‐related activities or strategies that were employed.

The conclusions drawn from the quantitative and qualitative syntheses will be combined to inform the review's final discussion and conclusions. The logic model will be revised based on the review's conclusions. The review's discussion will include reflections on the review's policy and future research implications.

#### 3.3.12 Subgroup analysis and investigation of heterogeneity

A formal statistical test will be used to test differences between outcomes. For subgroups defined by binary or nominal categories, we will use the Cochran *Q* test. For ordinal categories, multi‐level meta‐analysis will be conducted. The results of all subgroup testing will be reported, regardless of results. Results will be displayed using forest plots.

Should we have a sufficient number of included studies, we will conduct subgroup analyses by the following:

- Study duration (<6/≥6 months)
- Country income status (low income/middle‐income and upper‐middle income)
- Study design (RCT/NRS)
- Intervention type
  a. By opportunity structure dimension (economic, intrafamilial, sociocultural, legal, political)
  b. By nutrition‐related activity type (food supplementation, food fortification, micronutrient supplementation, etc.)

We will explore heterogeneity using preplanned subgroups and sensitivity analyses and report on the results. In cases where there is substantial variation in results between studies, especially where there is inconsistency in the direction of effect, we will not conduct a meta‐analysis and will use another form of synthesis (e.g., summary of effect estimates).

#### 3.3.13 Sensitivity analysis

Sensitivity analyses will be conducted to assess the impact of risk of bias (very low or low/moderate/high) and study design (randomized trials vs nonrandomized studies).

#### 3.3.14 Summary of findings and assessment of the certainty of the evidence

The level of evidence will be considered when formulating the review's conclusions. We will prepare GRADE summary of findings tables (Schunemann et al., 2013) for quantitative and qualitative evidence.

The GRADE process will be applied by two independent reviewers to all primary quantitative outcomes. An overall level of evidence quality (high, moderate, low, very low) for each primary outcome will be assigned as part of the GRADE process. Where possible, differences in results will be explained by giving a description of likely explanatory factors.

The GRADE‐CERQual approach will be used to assess the overall confidence in the qualitative evidence synthesis (Lewin et al., 2015). GRADE‐CERQual provides an assessment of confidence regarding the extent to which the research finding is likely to be substantially different from the phenomenon of interest. A level of confidence in the review findings will be assigned, ranging from high, moderate, low to very low confidence.

## CONTRIBUTIONS OF AUTHORS

- *Content*: Alison Riddle (AYR), Zulfiqar A. Bhutta (ZAB), Carol Vlassoff (CV), Elizabeth Kristjansson (EK), Abigail K. Ramage (AKR), Cynthia M. Kroeger (CMK), Anita Rizvi (AR), Alexandria Bennett (AB), Sara Wuehler (SW), George A. Wells (GAW), Vivian Welch (VW).
- *Systematic review methods*: GW, VW, EK.
- *Statistical analysis*: GAW, VW, MT.
- *Information retrieval*: Becky Skidmore (BS).

## DECLARATIONS OF INTEREST

AYR, AKR, EK, AR, AB, MT, BS, ZAB, CV, CK, and GAW have no conflicts of interest to declare. VW is the Editor‐in‐Chief of the Campbell Collaboration. SW is employed by Nutrition International (NI), an agency that implements nutrition programs for women and children. The findings of this review will be of value to NI, but employment at NI has no influence on the inputs that SW will provide to this review and NI has no desire to influence the outcomes of this review beyond good scientific process.

### PRELIMINARY TIMEFRAME

Approximate date for submission of the systematic review: December 31, 2021.

### PLANS FOR UPDATING THIS REVIEW

AYR will be responsible for updating this review.

## APPENDIX A SEARCH STRATEGY—OVID MEDLINE(R), EMBASE, PSYCINFO, EBM REVIEWS

First search conducted: 3 April 2019

Searched: Embase Classic+Embase <1947 to 2019 March 29 > , Ovid MEDLINE(R) ALL < 1946 to March 29, 2019 > , PsycINFO <1806 to March Week 4 2019 > , EBM Reviews ‐ Cochrane Central Register of Controlled Trials <February 2019 > , EBM Reviews ‐ Cochrane Database of Systematic Reviews <2005 to March 27, 2019 > , EBM Reviews ‐ Database of Abstracts of Reviews of Effects <1st Quarter 2016 > , EBM Reviews ‐ Health Technology Assessment <4th Quarter 2016 > , EBM Reviews ‐ NHS Economic Evaluation Database <1st Quarter 2016>

Search updated: 23 September 2020

Searched: Embase Classic+Embase <1947 to 2020 September 22 > , Ovid MEDLINE(R) ALL < 1946 to September 21, 2020 > , APA PsycInfo <1806 to September Week 2 2020 > , EBM Reviews ‐ Cochrane Central Register of Controlled Trials <August 2020 > , EBM Reviews ‐ Cochrane Database of Systematic Reviews <2005 to September 17, 2020>

1 "Power (Psychology)"/(158608)

2 Attitude/(107645)

3 Attitude to Health/(190744)

4 ((attitud* or opinion*) adj3 (diet* or eating or feeding or food? or health* or malnutrit* or malnourish* or meal? or nutrient? or nutrition or nutritive or snack?)).tw,kf. (41819)

5 Intention/(171233)

6 ((intention? or intent? or intend*) adj3 (diet* or eating or feeding or food? or health* or malnutrit* or malnourish* or meal? or nutrient? or nutrition or nutritive or snack?)).tw,kf. (8807)

7 Motivation/(216063)

8 (motivat* adj3 (diet* or eating or feeding or food? or health* or malnutrit* or malnourish* or meal? or nutrient? or nutrition or nutritive or snack?)).tw,kf. (13802)

9 (encourag* adj3 (diet* or eating or feeding or food? or health* or malnutrit* or malnourish* or meal? or nutrient? or nutrition or nutritive or snack?)).tw,kf. (14391)

10 Self Efficacy/(92885)

11 Personal Autonomy/(29331)

12 Self Concept/(184924)

13 (capable or capabilit* or emancipat* or empower* or agency or self‐efficac* or power* or autonomy or (self adj2 determin*) or (self adj2 concept*) or (self adj2 confiden*) or (self adj2 percept*) or (self adj2 esteem)).tw,kf. (2505725)

14 (abilit* adj3 (act or acted or acting or acts or action?)).tw,kf. (7288)

15 Women's Rights/(15820)

16 ((wom#n* adj2 right?) or (wom#n* adj2 status*) or (girl* adj2 right?) or (girl* adj2 status*) or (female? adj2 right?) or (female? adj2 status*) or (wife adj2 right?) or (wife adj2 status*) or (mother* adj2 right?) or (mother* adj2 status*) or (wives adj2 right?) or (wives adj2 status*)).tw,kf. (36459)

17 ((wom#n* or girl* or female? or mother* or wife or wives) adj2 (engag* or involv* or participat*)).tw,kf. (74261)

18 (active$2 adj2 (engag* or involv* or participat*)).tw,kf. (61724)

19 participatory.tw,kf. (35824)

20 ((wom#n* or girl* or female? or mother* or wife or wives) adj3 (advoca* or input* or ombuds* or represent*)).tw,kf. (21145)

21 ((citizen* or communit* or gender* or public or village?) adj3 (advoca* or input* or ombuds* or represent*)).tw,kf. (31601)

22 Gender Identity/(41227)

23 ((wom#n* or girl* or female? or mother* or wife or wives) adj3 role?).tw,kf. (28904)

24 Social Norms/(10338)

25 ((social* or societ* or cultural* or group?) adj3 (custom* or norm?)).tw,kf. (36015)

26 or/1‐25 (3571314)

27 Nutritional Requirements/(37442)

28 Nutritional Status/(105385)

29 Nutritive Value/(30809)

30 Adolescent Nutritional Physiological Phenomena/(1838)

31 Maternal Nutritional Physiological Phenomena/(13721)

32 Malnutrition/(75398)

33 exp Deficiency Diseases/(260675)

34 (avitaminos#s or hypovitaminos#s or hypo‐vitaminos#s).tw,kf. (8831)

35 ((ascorbic acid? or ferrous ascorbate or hybrin or magnesium ascorbate or magnesium ascorbicum or magnesium di‐L‐ascorbate or magnorbin or sodium ascorbate) adj3 (deficien* or deficit? or inadequa* or insufficien* or intak* or lack* or supplement*)).tw,kf. (4094)

36 scurvy.tw,kf. (4345)

37 ((all‐trans‐retinol or aquasol a or retinol) adj3 (deficien* or deficit? or inadequa* or insufficien* or intak* or lack* or supplement*)).tw,kf. (1959)

38 ((bursine or choline or fagine or vagine) adj3 (deficien* or deficit? or inadequa* or insufficien* or intak* or lack* or supplement*)).tw,kf. (7851)

39 ((folacin or folate or folic acid? or folvite or IFA or pteroylglutamic acid?) adj3 (deficien* or deficit? or inadequa* or insufficien* or intak* or lack*or supplement*)).tw,kf. (17888)

40 (hyperhomocystein?emi* or hyper‐homocystein?emi*).tw,kf. (15262)

41 ((niacin? or enduracin or induracin or lithium nicotinate or nicamin or nico‐400 or nicobid or nicocap or nicolar or nicotinate or nicotinic acid? or wampocap) adj3 (deficien* or deficit? or inadequa* or insufficien* or intak* or lack* or supplement*)).tw,kf. (1819)

42 ((tryptophan or ardeydorm or ardeytropin or L‐tryptophan or levotryptophan or lyphan or naturruhe or optimax or pms‐tryptophan or trofan or tryptacin or tryptan or ratio‐tryptophan) adj3 (deficien* or deficit? or inadequa* or insufficien* or intak* or lack* or supplement*)).tw,kf. (2802)

43 pellagra.tw,kf. (2774)

44 (riboflavin? adj3 (deficien* or deficit? or inadequa* or insufficien* or intak* or lack* or supplement*)).tw,kf. (3255)

45 ((thiamine? or aneurin or thiamin) adj3 (deficien* or deficit? or inadequa* or insufficien* or intak* or lack* or supplement*)).tw,kf. (7920)

46 (pyridoxine? adj3 (deficien* or deficit? or inadequa* or insufficien* or intak* or lack* or supplement*)).tw,kf. (2773)

47 Overnutrition/(5853)

48 exp Obesity/(711110)

49 Growth Disorders/(26038)

50 ((growth adj2 stunt$2) or stunting).tw,kf. (14027)

51 (nutrient? or nutrition* or nutritive or nourish* or malnutrit* or malnourish* or undernutrition or undernourish* or overnutrition or overnourish* or obese or obesity or wasting or underweight or under weight or overweight or over weight).tw,kf. (1769168)

52 Anemia/(242868)

53 exp Anemia, Hypochromic/(46742)

54 (an?emic or an?emia* or chloros#s).tw,kf. (387455)

55 Feeding Behavior/(172106)

56 ((eating or feeding or food or meal? or snack*) adj3 (behavio?r* or habit? or habitual* or pattern* or practice?)).tw,kf. (141785)

57 Diet/(402699)

58 (diet or dieted or diets or dietary or dieting).tw,kf. (1174789)

59 Energy Intake/(93531)

60 ((calori* or energy or protein?) adj3 intak*).tw,kf. (115306)

61 Healthy Diet/(4701)

62 ((eating or feeding or food? or meal? or snack*) adj3 (healthy or unhealthy)).tw,kf. (39237)

63 exp Micronutrients/(688381)

64 (micronutri* or micro‐nutri*).tw,kf. (35383)

65 (trace element? or trace mineral? or micromineral? or micro‐mineral?).tw,kf. (44266)

66 Calcium, Dietary/(31557)

67 Chromium/(59565)

68 Copper/(178475)

69 Iodine/(75382)

70 Iron/(259984)

71 Manganese/(74289)

72 Molybdenum/(21257)

73 Selenium/(58025)

74 Zinc/(170532)

75 (calcium or chromium or copper or fluoride or iodine or iron or manganese or molybdenum or selenium or zinc).tw,kf. (1924054)

76 vitamin*.tw,kf. (512331)

77 exp Dietary Supplements/(89126)

78 ((eating or feeding or food? or meal? or snack*) adj3 supplement*).tw,kf. (25906)

79 n?utr#ceutical?.tw,kf. (14005)

80 Food, Fortified/(11093)

81 ((fortif* or enrich* or functional) adj2 (eating or feeding or food? or meal? or snack*)).tw,kf. (25026)

82 ((home? or school* or work*) adj2 (ate or eaten or eating or eats or breakfast* or lunch* or dinner* or supper* or feed* or food? or meal?)).tw,kf. (19850)

83 Food Supply/(28920)

84 ((eating or feeding or food? or meal? or snack*) adj3 (secure* or securit* or insecure* or insecurit* or supply* or supplie*)).tw,kf. (31860)

85 or/27‐84 (6032125)

86 26 and 85 (298093)

87 Developing Countries/(159704)

88 Africa/or Asia/or Caribbean/or West Indies/or South America/or Latin America/or Central America/(228463)

89 (Afghanistan or Albania or Algeria or Angola or Argentina or Armenia or Armenian or Azerbaijan or Bangladesh or Benin or Byelarus or Byelorussian or Belarus or Belorussian or Belorussia or Belize or Bhutan or Bolivia or Bosnia or Herzegovina or Hercegovina or Botswana or Brazil or Bulgaria or Burkina Faso or Burkina Fasso or Upper Volta or Burundi or Urundi or Cambodia or Khmer Republic or Kampuchea or Cameroon or Cameroons or Cameron or Camerons or Cape Verde or Central African Republic or Chad or China or Colombia or Comoros or Comoro Islands or Comores or Mayotte or Congo or Zaire or Costa Rica or Cote d'Ivoire or Ivory Coast or Cuba or Djibouti or French Somaliland or Dominica or Dominican Republic or East Timor or East Timur or Timor Leste or Ecuador or Egypt or United Arab Republic or El Salvador or Eritrea or Ethiopia or Fiji or Gabon or Gabonese Republic or Gambia or Gaza or Georgia Republic or Georgian Republic or Ghana or Grenada or Guatemala or Guinea or Guiana or Guyana or Haiti or Honduras or India or Maldives or Indonesia or Iran or Iraq or Jamaica or Jordan or Kazakhstan or Kazakh or Kenya or Kiribati or Korea or Kosovo or Kyrgyzstan or Kirghizia or Kyrgyz Republic or Kirghiz or Kirgizstan or Lao PDR or Laos or Lebanon or Lesotho or Basutoland or Liberia or Libya or Macedonia or Madagascar or Malagasy Republic or Malaysia or Malaya or Malay or Sabah or Sarawak or Malawi or Mali or Marshall Islands or Mauritania or Mauritius or Agalega Islands or Mexico or Micronesia or Middle East or Moldova or Moldovia or Moldovian or Mongolia or Montenegro or Morocco or Ifni or Mozambique or Myanmar or Myanma or Burma or Namibia or Nepal or Netherlands Antilles or Nicaragua or Niger or Nigeria or Muscat or Pakistan or Palau or Palestine or Panama or Paraguay or Peru or Philippines or Philipines or Phillipines or Phillippines or Papua New Guinea or Romania or Rumania or Roumania or Rwanda or Ruanda or Saint Lucia or St Lucia or Saint Vincent or St Vincent or Grenadines or Samoa or Samoan Islands or Navigator Island or Navigator Islands or Sao Tome or Senegal or Serbia or Montenegro or Seychelles or Sierra Leone or Sri Lanka or Solomon Islands or Somalia or Sudan or Suriname or Surinam or Swaziland or South Africa or Syria or Tajikistan or Tadzhikistan or Tadjikistan or Tadzhik or Tanzania or Thailand or Togo or Togolese Republic or Tonga or Tunisia or Turkey or Turkmenistan or Turkmen or Uganda or Ukraine or Uzbekistan or Uzbek or Vanuatu or New Hebrides or Venezuela or Vietnam or Viet Nam or West Bank or Yemen or Zambia or Zimbabwe).tw,kf. (2468939)

90 exp africa/or exp africa, northern/or algeria/or egypt/or libya/or morocco/or tunisia/or exp "africa south of the sahara"/or africa, central/or cameroon/or central african republic/or chad/or congo/or "democratic republic of the congo"/or equatorial guinea/or gabon/or africa, eastern/or burundi/or djibouti/or eritrea/or ethiopia/or kenya/or rwanda/or somalia/or south sudan/or sudan/or tanzania/or uganda/or africa, southern/or angola/or botswana/or lesotho/or malawi/or mozambique/or namibia/or south africa/or swaziland/or zambia/or zimbabwe/or africa, western/or benin/or burkina faso/or cape verde/or cote d'ivoire/or gambia/or ghana/or guinea/or guinea‐bissau/or liberia/or mali/or mauritania/or niger/or nigeria/or senegal/or sierra leone/or togo/or americas/or exp caribbean region/or exp west indies/or exp central america/or belize/or costa rica/or el salvador/or guatemala/or honduras/or nicaragua/or panama/or panama canal zone/or latin america/or mexico/or exp south america/or argentina/or bolivia/or brazil/or chile/or colombia/or ecuador/or french guiana/or guyana/or paraguay/or peru/or suriname/or uruguay/or venezuela/or asia/or asia, central/or kazakhstan/or kyrgyzstan/or tajikistan/or turkmenistan/or uzbekistan/or exp asia, southeastern/or borneo/or brunei/or cambodia/or timor‐leste/or indonesia/or laos/or malaysia/or mekong valley/or myanmar/or philippines/or singapore/or thailand/or vietnam/or asia, western/or bangladesh/or bhutan/or india/or sikkim/or middle east/or afghanistan/or bahrain/or iran/or iraq/or israel/or jordan/or kuwait/or lebanon/or oman/or qatar/or saudi arabia/or syria/or turkey/or united arab emirates/or yemen/or nepal/or pakistan/or sri lanka/or far east/or china/or beijing/or macau/or tibet/or korea/or mongolia/or taiwan/or indian ocean islands/or comoros/or madagascar/or mauritius/or reunion/or seychelles/or pacific islands/or exp melanesia/or exp micronesia/or polynesia/or pitcairn island/or exp samoa/or tonga/or prince edward island/or west indies/or "antigua and barbuda"/or bahamas/or barbados/or cuba/or dominica/or dominican republic/or grenada/or guadeloupe/or haiti/or jamaica/or martinique/or netherlands antilles/or puerto rico/or "saint kitts and nevis"/or saint lucia/or "saint vincent and the grenadines"/or "trinidad and tobago"/or united states virgin islands/or oceania/(2424512)

91 ((developing or less* developed or under developed or underdeveloped or middle income or low* income or underserved or under served or deprived or poor*) adj (countr* or nation? or population? or region? or world or state*)).tw,kf. (262888)

92 ((developing or less* developed or under developed or underdeveloped or middle income or low* income or poor*) adj econom*).tw,kf. (3030)

93 (low* adj (gdp or gnp or gross domestic or gross national)).tw,kf. (641)

94 (low adj3 middle adj3 countr*).tw,kf. (31752)

95 (lmic or lmics or third world or lami countr*).tw,kf. (16026)

96 transitional countr*.tw,kf. (457)

97 or/87‐96 (3718691)

98 86 and 97 (32745)

99 Women/(8405706)

100 Pregnant Women/(69649)

101 Female/and (Adolescent/or Adult/or Young Adult/) (9600899)

102 (woman* or women* or matern* or mother* or wife or wives or pregnan*).tw,kf. (4328988)

103 ((female? or girl*) adj5 (adolescen* or adult* or child‐bearing or childbearing or highschool* or school* or teen* or marry* or marriage* or marrie? or menstru* or post‐pubescen* or postpubescen* or post‐puberty or postpuberty or reproductive or spous* or youth* or young adult?)).tw,kf. (286629)

104 ((female? or girl or girls) adj2 (ten year? or ten yr? or 10 year? or 10 yr? or eleven year? or eleven yr? or 11 year? or 11 yr? or twelve year? or twelve yr? or 12 year? or 12 yr?)).tw,kf. (28995)

105 ((female? or girl or girls) adj2 (age? or old) adj3 ("10" or ten or "11" or eleven or "12" or twelve)).tw,kf. (66012)

106 young girl?.tw,kf. (10028)

107 or/99‐106 (15081858)

108 98 and 107 (19470)

109 exp Animals/not Humans/(17546303)

110 108 not 109 [ANIMAL‐ONLY REMOVED] (13985)

111 (comment or editorial or news or newspaper article).pt. (1896536)

112 (letter not (letter and randomized controlled trial)).pt. (2072075)

113 110 not (111 or 112) [OPINION PIECES REMOVED] (13938)

114 113 use medall [MEDLINE RECORDS] (6474)

115 empowerment/(27108)

116 attitude/(107645)

117 attitude to health/(190744)

118 attitude to life/(657)

119 maternal attitude/(2423)

120 attitude to pregnancy/(580)

121 social attitude/(2312)

122 ((attitud* or opinion*) adj3 (diet* or eating or feeding or food? or health* or malnutrit* or malnourish* or meal? or nutrient? or nutrition or nutritive or snack?)).tw,kw. (45396)

123 behavior/(209991)

124 ((intention? or intent? or intend*) adj3 (diet* or eating or feeding or food? or health* or malnutrit* or malnourish* or meal? or nutrient? or nutrition or nutritive or snack?)).tw,kw. (9069)

125 motivation/(216063)

126 (motivat* adj3 (diet* or eating or feeding or food? or health* or malnutrit* or malnourish* or meal? or nutrient? or nutrition or nutritive or snack?)).tw,kw. (14513)

127 (encourag* adj3 (diet* or eating or feeding or food? or health* or malnutrit* or malnourish* or meal? or nutrient? or nutrition or nutritive or snack?)).tw,kw. (14391)

128 personal autonomy/(29331)

129 exp self concept/(373341)

130 (capable or capabilit* or emancipat* or empower* or agency or self‐efficac* or power* or autonomy or (self adj2 determin*) or (self adj2 concept*) or (self adj2 confiden*) or (self adj2 percept*) or (self adj2 esteem)).tw,kw. (2512016)

131 (abilit* adj3 (act or acted or acting or acts or action?)).tw,kw. (7289)

132 women's rights/(15820)

133 ((wom#n* adj2 right?) or (wom#n* adj2 status*) or (girl* adj2 right?) or (girl* adj2 status*) or (female? adj2 right?) or (female? adj2 status*) or (wife adj2 right?) or (wife adj2 status*) or (mother* adj2 right?) or (mother* adj2 status*) or (wives adj2 right?) or (wives adj2 status*)).tw,kw. (36781)

134 ((wom#n* or girl* or female? or mother* or wife or wives) adj2 (engag* or involv* or participat*)).tw,kw. (74297)

135 (active$2 adj2 (engag* or involv* or participat*)).tw,kw. (61734)

136 participatory*.tw,kw. (36423)

137 ((wom#n* or girl* or female? or mother* or wife or wives) adj3 (advoca* or input* or ombuds* or represent*)).tw,kw. (21169)

138 ((citizen* or communit* or gender* or public or village?) adj3 (advoca* or input* or ombuds* or represent*)).tw,kw. (31657)

139 gender identity/(41227)

140 ((wom#n* or girl* or female? or mother* or wife or wives) adj3 role?).tw,kw. (28723)

141 social norm/(10891)

142 ((social* or societ* or cultural* or group?) adj3 (custom* or norm?)).tw,kw. (36159)

143 or/115‐142 (3718206)

144 nutrition/(124564)

145 nutritional health/(6190)

146 exp nutritional requirement/(39461)

147 exp nutritional status/(106035)

148 nutritional value/(31912)

149 adolescent nutrition/(202)

150 maternal nutrition/(11173)

151 malnutrition/(75398)

152 exp nutritional deficiency/(283978)

153 exp protein deficiency/(44783)

154 (avitaminos#s or hypovitaminos#s or hypo‐vitaminos#s).tw,kw. 5((ascorbic acid? or ferrous ascorbate or hybrin or magnesium ascorbate or magnesium ascorbicum or magnesium di‐L‐ascorbate or magnorbin or sodium ascorbate) adj3 (deficien* or deficit? or inadequa* or insufficien* or intak* or lack* or supplement*)).tw,kw. (4087)

156 scurvy.tw,kw. (4353)

157 ((all‐trans‐retinol or aquasol a or retinol) adj3 (deficien* or deficit? or inadequa* or insufficien* or intak* or lack* or supplement*)).tw,kw. (2055)

158 ((bursine or choline or fagine or vagine) adj3 (deficien* or deficit? or inadequa* or insufficien* or intak* or lack* or supplement*)).tw,kw. (7851)

159 ((folacin or folate or folic acid? or folvite or IFA or pteroylglutamic acid?) adj3 (deficien* or deficit? or inadequa* or insufficien* or intak* or lack*or supplement*)).tw,kw. (17918)

160 (hyperhomocystein?emi* or hyper‐homocystein?emi*).tw,kw. (15531)

161 ((niacin? or enduracin or induracin or lithium nicotinate or nicamin or nico‐400 or nicobid or nicocap or nicolar or nicotinate or nicotinic acid? or wampocap) adj3 (deficien* or deficit? or inadequa* or insufficien* or intak* or lack* or supplement*)).tw,kw. (1816)

162 ((tryptophan or ardeydorm or ardeytropin or L‐tryptophan or levotryptophan or lyphan or naturruhe or optimax or pms‐tryptophan or trofan or tryptacin or tryptan or ratio‐tryptophan) adj3 (deficien* or deficit? or inadequa* or insufficien* or intak* or lack* or supplement*)).tw,kw. (2805)

163 pellagra.tw,kw. (2772)

164 (riboflavin? adj3 (deficien* or deficit? or inadequa* or insufficien* or intak* or lack* or supplement*)).tw,kw. (3220)

165 ((thiamine? or aneurin or thiamin) adj3 (deficien* or deficit? or inadequa* or insufficien* or intak* or lack* or supplement*)).tw,kw. (7929)

166 (pyridoxine? adj3 (deficien* or deficit? or inadequa* or insufficien* or intak* or lack* or supplement*)).tw,kw. (2690)

167 exp overnutrition/(694990)

168 growth disorder/(33286)

169 stunting/(19313)

170 ((growth adj2 stunt$2) or stunting).tw,kw. (14278)

171 (nutrient? or nutrition* or nutritive or nourish* or malnutrit* or malnourish* or undernutrition or undernourish* or overnutrition or overnourish* or obese or obesity or wasting or underweight or under weight or overweight or over weight).tw,kw. (1793960)

172 anemia/(242868)

173 iron deficiency anemia/(37358)

174 (an?emic or an?emia* or chloros#s).tw,kw. (395051)

175 eating habit/(99906)

176 feeding behavior/(172106)

177 ((eating or feeding or food or meal? or snack*) adj3 (behavio?r* or habit? or habitual* or pattern* or practice?)).tw,kw. (144241)

178 diet/(402699)

179 (diet or dieted or diets or dietary or dieting).tw,kw. (1187821)

180 caloric intake/(96310)

181 ((calori* or energy or protein?) adj3 intak*).tw,kw. (117328)

182 dietary intake/(73687)

183 healthy diet/(4701)

184 ((eating or feeding or food? or meal? or snack*) adj3 (healthy or unhealthy)).tw,kw. (39335)

185 exp nutrient/(677172)

186 nutrient availability/(3222)

187 nutrient content/(5017)

188 nutrient limitation/(1029)

189 nutrient supply/(2308)

190 nutrient uptake/(2874)

191 (micronutri* or micro‐nutri*).tw,kw. (36132)

192 exp trace element/(366886)

193 (trace element? or trace mineral? or micromineral? or micro‐mineral?).tw,kw. (46036)

194 chromium/(59565)

195 copper/(178475)

196 iodine/(75382)

197 manganese/(74289)

198 molybdenum/(21257)

199 selenium/(58025)

200 zinc/(170532)

201 (calcium or chromium or copper or fluoride or iodine or iron or manganese or molybdenum or selenium or zinc).tw,kw. (1949030)

202 vitamin/(89429)

203 vitamin*.tw,kw. (522115)

204 dietary supplement/(61637)

205 ((eating or feeding or food? or meal? or snack*) adj3 supplement*).tw,kw. (26015)

206 nutraceutical/(64077)

207 n?utr#ceutical?.tw,kw. (14562)

208 fortified food/(11096)

209 ((fortif* or enrich* or functional) adj2 (eating or feeding or food? or meal? or snack*)).tw,kw. (25657)

210 ((home? or school* or work*) adj2 (ate or eaten or eating or eats or breakfast* or lunch* or dinner* or supper* or feed* or food? or meal?)).tw,kw. (20058)

211 food availability/(3871)

212 ((eating or feeding or food? or meal? or snack*) adj3 (secure* or securit* or insecure* or insecurit* or supply* or supplie*)).tw,kw. (31599)

213 or/144‐212 (6081563)

214 143 and 213 (313876)

215 developing country/(165162)

216 (Africa or Asia or Caribbean or West Indies or South America or Latin America or Central America).hw,tw,kw. (640442)

217 (Afghanistan or Albania or Algeria or Angola or Argentina or Armenia or Armenian or Azerbaijan or Bangladesh or Benin or Byelarus or Byelorussian or Belarus or Belorussian or Belorussia or Belize or Bhutan or Bolivia or Bosnia or Herzegovina or Hercegovina or Botswana or Brazil or Bulgaria or Burkina Faso or Burkina Fasso or Upper Volta or Burundi or Urundi or Cambodia or Khmer Republic or Kampuchea or Cameroon or Cameroons or Cameron or Camerons or Cape Verde or Central African Republic or Chad or China or Colombia or Comoros or Comoro Islands or Comores or Mayotte or Congo or Zaire or Costa Rica or Cote d'Ivoire or Ivory Coast or Cuba or Djibouti or French Somaliland or Dominica or Dominican Republic or East Timor or East Timur or Timor Leste or Ecuador or Egypt or United Arab Republic or El Salvador or Eritrea or Ethiopia or Fiji or Gabon or Gabonese Republic or Gambia or Gaza or Georgia Republic or Georgian Republic or Ghana or Grenada or Guatemala or Guinea or Guiana or Guyana or Haiti or Honduras or India or Maldives or Indonesia or Iran or Iraq or Jamaica or Jordan or Kazakhstan or Kazakh or Kenya or Kiribati or Korea or Kosovo or Kyrgyzstan or Kirghizia or Kyrgyz Republic or Kirghiz or Kirgizstan or Lao PDR or Laos or Lebanon or Lesotho or Basutoland or Liberia or Libya or Macedonia or Madagascar or Malagasy Republic or Malaysia or Malaya or Malay or Sabah or Sarawak or Malawi or Mali or Marshall Islands or Mauritania or Mauritius or Agalega Islands or Mexico or Micronesia or Middle East or Moldova or Moldovia or Moldovian or Mongolia or Montenegro or Morocco or Ifni or Mozambique or Myanmar or Myanma or Burma or Namibia or Nepal or Netherlands Antilles or Nicaragua or Niger or Nigeria or Muscat or Pakistan or Palau or Palestine or Panama or Paraguay or Peru or Philippines or Philipines or Phillipines or Phillippines or Papua New Guinea or Romania or Rumania or Roumania or Rwanda or Ruanda or Saint Lucia or St Lucia or Saint Vincent or St Vincent or Grenadines or Samoa or Samoan Islands or Navigator Island or Navigator Islands or Sao Tome or Senegal or Serbia or Montenegro or Seychelles or Sierra Leone or Sri Lanka or Solomon Islands or Somalia or Sudan or Suriname or Surinam or Swaziland or South Africa or Syria or Tajikistan or Tadzhikistan or Tadjikistan or Tadzhik or Tanzania or Thailand or Togo or Togolese Republic or Tonga or Tunisia or Turkey or Turkmenistan or Turkmen or Uganda or Ukraine or Uzbekistan or Uzbek or Vanuatu or New Hebrides or Venezuela or Vietnam or Viet Nam or West Bank or Yemen or Zambia or Zimbabwe).hw,tw,kw. (3320872)

218 exp africa/or exp africa, northern/or algeria/or egypt/or libya/or morocco/or tunisia/or exp "africa south of the sahara"/or africa, central/or cameroon/or central african republic/or chad/or congo/or "democratic republic of the congo"/or equatorial guinea/or gabon/or africa, eastern/or burundi/or djibouti/or eritrea/or ethiopia/or kenya/or rwanda/or somalia/or south sudan/or sudan/or tanzania/or uganda/or africa, southern/or angola/or botswana/or lesotho/or malawi/or mozambique/or namibia/or south africa/or swaziland/or zambia/or zimbabwe/or africa, western/or benin/or burkina faso/or cape verde/or cote d'ivoire/or gambia/or ghana/or guinea/or guinea‐bissau/or liberia/or mali/or mauritania/or niger/or nigeria/or senegal/or sierra leone/or togo/or americas/or exp caribbean region/or exp west indies/or exp central america/or belize/or costa rica/or el salvador/or guatemala/or honduras/or nicaragua/or panama/or panama canal zone/or latin america/or mexico/or exp south america/or argentina/or bolivia/or brazil/or chile/or colombia/or ecuador/or french guiana/or guyana/or paraguay/or peru/or suriname/or uruguay/or venezuela/or asia/or asia, central/or kazakhstan/or kyrgyzstan/or tajikistan/or turkmenistan/or uzbekistan/or exp asia, southeastern/or borneo/or brunei/or cambodia/or timor‐leste/or indonesia/or laos/or malaysia/or mekong valley/or myanmar/or philippines/or singapore/or thailand/or vietnam/or asia, western/or bangladesh/or bhutan/or india/or sikkim/or middle east/or afghanistan/or bahrain/or iran/or iraq/or israel/or jordan/or kuwait/or lebanon/or oman/or qatar/or saudi arabia/or syria/or turkey/or united arab emirates/or yemen/or nepal/or pakistan/or sri lanka/or far east/or china/or beijing/or macau/or tibet/or korea/or mongolia/or taiwan/or indian ocean islands/or comoros/or madagascar/or mauritius/or reunion/or seychelles/or pacific islands/or exp melanesia/or exp micronesia/or polynesia/or pitcairn island/or exp samoa/or tonga/or prince edward island/or west indies/or "antigua and barbuda"/or bahamas/or barbados/or cuba/or dominica/or dominican republic/or grenada/or guadeloupe/or haiti/or jamaica/or martinique/or netherlands antilles/or puerto rico/or "saint kitts and nevis"/or saint lucia/or "saint vincent and the grenadines"/or "trinidad and tobago"/or united states virgin islands/or oceania/(2424512)

219 ((developing or less* developed or under developed or underdeveloped or middle income or low* income or underserved or under served or deprived or poor*) adj (countr* or nation? or population? or region? or world or state*)).tw,kw. (237596)

220 ((developing or less* developed or under developed or underdeveloped or middle income or low* income or poor*) adj econom*).tw,kw. (3036)

221 (low* adj (gdp or gnp or gross domestic or gross national)).tw,kw. (645)

222 (low adj3 middle adj3 countr*).tw,kw. (31748)

223 (lmic or lmics or third world or lami countr*).tw,kw. (16181)

224 transitional countr*.tw,kw. (462)

225 or/215‐224 (4044608)

226 214 and 225 (36165)

227 exp named groups by pregnancy/(101747)

228 pregnant woman/(82942)

229 female/and (adult/or young adult/or juvenile/or adolescent/or adolescent parent/) (9605834)

230 (woman* or women* or matern* or mother* or wife or wives or pregnan*).tw,kw. (4340623)

231 ((female? or girl*) adj5 (adolescen* or adult* or child‐bearing or childbearing or highschool* or school* or teen* or marry* or marriage* or marrie? or menstru* or post‐pubescen* or postpubescen* or post‐puberty or postpuberty or reproductive or spous* or youth* or young adult?)).tw,kw. (292884)

232 ((female? or girl or girls) adj2 (ten year? or ten yr? or 10 year? or 10 yr? or eleven year? or eleven yr? or 11 year? or 11 yr? or twelve year? or twelve yr? or 12 year? or 12 yr?)).tw,kw. (28995)

233 ((female? or girl or girls) adj2 (age? or old) adj3 ("10" or ten or "11" or eleven or "12" or twelve)).tw,kw. (66013)

234 young girl?.tw,kw. (10038)

235 or/227‐234 (12506222)

236 226 and 235 (20195)

237 exp animal/or exp animal experimentation/or exp animal model/or exp animal experiment/or nonhuman/or exp vertebrate/(50805119)

238 exp human/or exp human experimentation/or exp human experiment/(38981083)

239 237 not 238 (11825722)

240 236 not 239 [ANIMAL‐ONLY REMOVED] (20036)

241 editorial.pt. (1081172)

242 letter.pt. not (letter.pt. and randomized controlled trial/) (2071940)

243 240 not (241 or 242) [OPINION PIECES REMOVED] (19985)

244 243 use emczd [EMBASE RECORDS] (9903)

245 Social Identity/(105394)

246 Group Identity/(1322)

247 Attitudes/(72109)

248 Community Attitudes/(2559)

249 Death Attitudes/(19039)

250 Eating Attitudes/(1539)

251 Female Attitudes/(1625)

252 Health Attitudes/(94051)

253 exp Sex Role Attitudes/(12044)

254 ((attitud* or opinion*) adj3 (diet* or eating or feeding or food? or health* or malnutrit* or malnourish* or meal? or nutrient? or nutrition or nutritive or snack?)).tw. (41335)

255 exp Intention/(3953989)

256 ((intention? or intent? or intend*) adj3 (diet* or eating or feeding or food? or health* or malnutrit* or malnourish* or meal? or nutrient? or nutrition or nutritive or snack?)).tw. (8802)

257 Motivation/(216063)

258 Expectations/(81852)

259 (motivat* adj3 (diet* or eating or feeding or food? or health* or malnutrit* or malnourish* or meal? or nutrient? or nutrition or nutritive or snack?)).tw. (13785)

260 (encourag* adj3 (diet* or eating or feeding or food? or health* or malnutrit* or malnourish* or meal? or nutrient? or nutrition or nutritive or snack?)).tw. (14391)

261 Self‐Efficacy/(92885)

262 "Independence (Personality)"/(4988)

263 Self‐Determination/(20619)

264 Self‐Concept/(184924)

265 Self‐Confidence/(57437)

266 Self‐Esteem/(101452)

267 (capable or capabilit* or emancipat* or empower* or agency or self‐efficac* or power* or autonomy or (self adj2 determin*) or (self adj2 concept*) or (self adj2 confiden*) or (self adj2 percept*) or (self adj2 esteem)).tw. (2502151)

268 (abilit* adj3 (act or acted or acting or acts or action?)).tw. (7288)

269 Self‐Perception/(146381)

270 ((wom#n* adj2 right?) or (wom#n* adj2 status*) or (girl* adj2 right?) or (girl* adj2 status*) or (female? adj2 right?) or (female? adj2 status*) or (wife adj2 right?) or (wife adj2 status*) or (mother* adj2 right?) or (mother* adj2 status*) or (wives adj2 right?) or (wives adj2 status*)).tw. (34478)

271 Involvement/(6748)

272 Participation/(7668)

273 ((wom#n* or girl* or female? or mother* or wife or wives) adj2 (engag* or involv* or participat*)).tw. (74248)

274 (active$2 adj2 (engag* or involv* or participat*)).tw. (61718)

275 participatory*.tw. (35257)

276 ((wom#n* or girl* or female? or mother* or wife or wives) adj3 (advoca* or input* or ombuds* or represent*)).tw. (21143)

277 ((citizen* or communit* or gender* or public or village?) adj3 (advoca* or input* or ombuds* or represent*)).tw. (31568)

278 Gender Equality/(667)

279 Role Expectations/(1621)

280 Sex Roles/(34691)

281 ((wom#n* or girl* or female? or mother* or wife or wives) adj3 role?).tw. (28694)

282 Social Norms/(10338)

283 ((social* or societ* or cultural* or group?) adj3 (custom* or norm?)).tw. (35820)

284 or/245‐283 (6964179)

285 Nutrition/(124564)

286 exp Nutritional Deficiencies/(124075)

287 (avitaminos#s or hypovitaminos#s or hypo‐vitaminos#s).tw. (8621)

288 ((ascorbic acid? or ferrous ascorbate or hybrin or magnesium ascorbate or magnesium ascorbicum or magnesium di‐L‐ascorbate or magnorbin or sodium ascorbate) adj3 (deficien* or deficit? or inadequa* or insufficien* or intak* or lack* or supplement*)).tw. (3989)

289 scurvy.tw. (3901)

290 ((all‐trans‐retinol or aquasol a or retinol) adj3 (deficien* or deficit? or inadequa* or insufficien* or intak* or lack* or supplement*)).tw. (1959)

291 ((bursine or choline or fagine or vagine) adj3 (deficien* or deficit? or inadequa* or insufficien* or intak* or lack* or supplement*)).tw. (7797)

292 ((folacin or folate or folic acid? or folvite or IFA or pteroylglutamic acid?) adj3 (deficien* or deficit? or inadequa* or insufficien* or intak* or lack*or supplement*)).tw. (17757)

293 (hyperhomocystein?emi* or hyper‐homocystein?emi*).tw. (15197)

294 ((niacin? or enduracin or induracin or lithium nicotinate or nicamin or nico‐400 or nicobid or nicocap or nicolar or nicotinate or nicotinic acid? or wampocap) adj3 (deficien* or deficit? or inadequa* or insufficien* or intak* or lack* or supplement*)).tw. (1792)

295 ((tryptophan or ardeydorm or ardeytropin or L‐tryptophan or levotryptophan or lyphan or naturruhe or optimax or pms‐tryptophan or trofan or tryptacin or tryptan or ratio‐tryptophan) adj3 (deficien* or deficit? or inadequa* or insufficien* or intak* or lack* or supplement*)).tw. (2784)

296 pellagra.tw. (2584)

297 (riboflavin? adj3 (deficien* or deficit? or inadequa* or insufficien* or intak* or lack* or supplement*)).tw. (3196)

298 ((thiamine? or aneurin or thiamin) adj3 (deficien* or deficit? or inadequa* or insufficien* or intak* or lack* or supplement*)).tw. (7817)

299 (pyridoxine? adj3 (deficien* or deficit? or inadequa* or insufficien* or intak* or lack* or supplement*)).tw. (2639)

300 Obesity/(598386)

301 ((growth adj2 stunt$2) or stunting).tw. (13942)

302 (nutrient? or nutrition* or nutritive or nourish* or malnutrit* or malnourish* or undernutrition or undernourish* or overnutrition or overnourish* or obese or obesity or wasting or underweight or under weight or overweight or over weight).tw. (1748835)

303 Anemia/(242868)

304 (an?emic or an?emia* or chloros#s).tw. (380030)

305 Eating Behavior/(174683)

306 ((eating or feeding or food or meal? or snack*) adj3 (behavio?r* or habit? or habitual* or pattern* or practice?)).tw. (141008)

307 Diets/(161532)

308 (diet or dieted or diets or dietary or dieting).tw. (1165001)

309 Food Intake/(184009)

310 ((calori* or energy or protein?) adj3 intak*).tw. (115118)

311 ((eating or feeding or food? or meal? or snack*) adj3 (healthy or unhealthy)).tw. (39122)

312 (micronutri* or micro‐nutri*).tw. (35029)

313 Metallic Elements/(436)

314 (trace element? or trace mineral? or micromineral? or micro‐mineral?).tw. (43646)

315 exp Calcium/(570689)

316 Copper/(178475)

317 Iron/(259984)

318 Zinc/(170532)

319 (calcium or chromium or copper or fluoride or iodine or iron or manganese or molybdenum or selenium or zinc).tw. (1900718)

320 Vitamins/(89507)

321 vitamin*.tw. (499855)

322 Dietary Supplements/(67852)

323 ((eating or feeding or food? or meal? or snack*) adj3 supplement*).tw. (25555)

324 n?utr#ceutical?.tw. (13721)

325 ((fortif* or enrich* or functional) adj2 (eating or feeding or food? or meal? or snack*)).tw. (24593)

326 ((home? or school* or work*) adj2 (ate or eaten or eating or eats or breakfast* or lunch* or dinner* or supper* or feed* or food? or meal?)).tw. (19806)

327 Food Deprivation/(18608)

328 ((eating or feeding or food? or meal? or snack*) adj3 (secure* or securit* or insecure* or insecurit* or supply* or supplie*)).tw. (30873)

329 or/285‐328 (5847359)

330 284 and 329 (656017)

331 Developing Countries/(159704)

332 (Africa? or Asia? or Caribbean or West Indies or South America? or Latin America? or Central America?).tw. (924816)

333 (Afghanistan or Albania or Algeria or Angola or Argentina or Armenia or Armenian or Azerbaijan or Bangladesh or Benin or Byelarus or Byelorussian or Belarus or Belorussian or Belorussia or Belize or Bhutan or Bolivia or Bosnia or Herzegovina or Hercegovina or Botswana or Brazil or Bulgaria or Burkina Faso or Burkina Fasso or Upper Volta or Burundi or Urundi or Cambodia or Khmer Republic or Kampuchea or Cameroon or Cameroons or Cameron or Camerons or Cape Verde or Central African Republic or Chad or China or Colombia or Comoros or Comoro Islands or Comores or Mayotte or Congo or Zaire or Costa Rica or Cote d'Ivoire or Ivory Coast or Cuba or Djibouti or French Somaliland or Dominica or Dominican Republic or East Timor or East Timur or Timor Leste or Ecuador or Egypt or United Arab Republic or El Salvador or Eritrea or Ethiopia or Fiji or Gabon or Gabonese Republic or Gambia or Gaza or Georgia Republic or Georgian Republic or Ghana or Grenada or Guatemala or Guinea or Guiana or Guyana or Haiti or Honduras or India or Maldives or Indonesia or Iran or Iraq or Jamaica or Jordan or Kazakhstan or Kazakh or Kenya or Kiribati or Korea or Kosovo or Kyrgyzstan or Kirghizia or Kyrgyz Republic or Kirghiz or Kirgizstan or Lao PDR or Laos or Lebanon or Lesotho or Basutoland or Liberia or Libya or Macedonia or Madagascar or Malagasy Republic or Malaysia or Malaya or Malay or Sabah or Sarawak or Malawi or Mali or Marshall Islands or Mauritania or Mauritius or Agalega Islands or Mexico or Micronesia or Middle East or Moldova or Moldovia or Moldovian or Mongolia or Montenegro or Morocco or Ifni or Mozambique or Myanmar or Myanma or Burma or Namibia or Nepal or Netherlands Antilles or Nicaragua or Niger or Nigeria or Muscat or Pakistan or Palau or Palestine or Panama or Paraguay or Peru or Philippines or Philipines or Phillipines or Phillippines or Papua New Guinea or Romania or Rumania or Roumania or Rwanda or Ruanda or Saint Lucia or St Lucia or Saint Vincent or St Vincent or Grenadines or Samoa or Samoan Islands or Navigator Island or Navigator Islands or Sao Tome or Senegal or Serbia or Montenegro or Seychelles or Sierra Leone or Sri Lanka or Solomon Islands or Somalia or Sudan or Suriname or Surinam or Swaziland or South Africa or Syria or Tajikistan or Tadzhikistan or Tadjikistan or Tadzhik or Tanzania or Thailand or Togo or Togolese Republic or Tonga or Tunisia or Turkey or Turkmenistan or Turkmen or Uganda or Ukraine or Uzbekistan or Uzbek or Vanuatu or New Hebrides or Venezuela or Vietnam or Viet Nam or West Bank or Yemen or Zambia or Zimbabwe).tw. (2449161)

334 ((developing or less* developed or under developed or underdeveloped or middle income or low* income or underserved or under served or deprived or poor*) adj (countr* or nation? or population? or region? or world or state*)).tw. (233745)

335 ((developing or less* developed or under developed or underdeveloped or middle income or low* income or poor*) adj econom*).tw. (3024)

336 (low* adj (gdp or gnp or gross domestic or gross national)).tw. (641)

337 (low adj3 middle adj3 countr*).tw. (31256)

338 (lmic or lmics or third world or lami countr*).tw. (15802)

339 transitional countr*.tw. (456)

340 or/331‐339 (3253265)

341 330 and 340 (74884)

342 Adolescent Mothers/(2282)

343 Expectant Mothers/(637)

344 (woman* or women* or matern* or mother* or wife or wives or pregnan*).tw. (4311022)

345 ((female? or girl*) adj5 (adolescen* or adult* or child‐bearing or childbearing or highschool* or school* or teen* or marry* or marriage* or marrie? or menstru* or post‐pubescen* or postpubescen* or post‐puberty or postpuberty or reproductive or spous* or youth* or young adult?)).tw. (285627)

346 ((female? or girl or girls) adj2 (ten year? or ten yr? or 10 year? or 10 yr? or eleven year? or eleven yr? or 11 year? or 11 yr? or twelve year? or twelve yr? or 12 year? or 12 yr?)).tw. (28995)

347 ((female? or girl or girls) adj2 (age? or old) adj3 ("10" or ten or "11" or eleven or "12" or twelve)).tw. (66012)

348 young girl?.tw. (10026)

349 or/342‐348 (4596550)

350 Human Females/(88344)

351 Wives/(13136)

352 350 or 351 (101298)

353 limit 352 to (200 adolescence <age 13 to 17 yrs> or 320 young adulthood <age 18 to 29 yrs> or 340 thirties <age 30 to 39 yrs> or 360 middle age <age 40 to 64 yrs > ) [Limit not valid in Embase,Ovid MEDLINE(R),Ovid MEDLINE(R) Daily Update,Ovid MEDLINE(R) In‐Process,Ovid MEDLINE(R) Publisher,CCTR,CDSR,DARE,CLHTA,CLEED; records were retained] (43270)

354 349 or 353 (4605043)

355 341 and 354 (27908)

356 355 use medall,emczd,cctr,coch,dare,clhta,cleed (26667)

357 355 not 356 [PSYCINFO RECORDS] (1241)

358 "Power (Psychology)"/(158608)

359 Attitude/(107645)

360 Attitude to Health/(190744)

361 ((attitud* or opinion*) adj3 (diet* or eating or feeding or food? or health* or malnutrit* or malnourish* or meal? or nutrient? or nutrition or nutritive or snack?)).ti,ab,kw. (42279)

362 Intention/(171233)

363 ((intention? or intent? or intend*) adj3 (diet* or eating or feeding or food? or health* or malnutrit* or malnourish* or meal? or nutrient? or nutrition or nutritive or snack?)).ti,ab,kw. (8886)

364 Motivation/(216063)

365 (motivat* adj3 (diet* or eating or feeding or food? or health* or malnutrit* or malnourish* or meal? or nutrient? or nutrition or nutritive or snack?)).ti,ab,kw. (14263)

366 (encourag* adj3 (diet* or eating or feeding or food? or health* or malnutrit* or malnourish* or meal? or nutrient? or nutrition or nutritive or snack?)).ti,ab,kw. (14226)

367 Self Efficacy/(92885)

368 Personal Autonomy/(29331)

369 Self Concept/(184924)

370 (capable or capabilit* or emancipat* or empower* or agency or self‐efficac* or power* or autonomy or (self adj2 determin*) or (self adj2 concept*) or (self adj2 confiden*) or (self adj2 percept*) or (self adj2 esteem)).ti,ab,kw. (2484054)

371 (abilit* adj3 (act or acted or acting or acts or action?)).ti,ab,kw. (7236)

372 Women's Rights/(15820)

373 ((wom#n* adj2 right?) or (wom#n* adj2 status*) or (girl* adj2 right?) or (girl* adj2 status*) or (female? adj2 right?) or (female? adj2 status*) or (wife adj2 right?) or (wife adj2 status*) or (mother* adj2 right?) or (mother* adj2 status*) or (wives adj2 right?) or (wives adj2 status*)).ti,ab,kw. (36005)

374 ((wom#n* or girl* or female? or mother* or wife or wives) adj2 (engag* or involv* or participat*)).ti,ab,kw. (73351)

375 (active$2 adj2 (engag* or involv* or participat*)).ti,ab,kw. (61322)

376 participatory*.ti,ab,kw. (35693)

377 ((wom#n* or girl* or female? or mother* or wife or wives) adj3 (advoca* or input* or ombuds* or represent*)).ti,ab,kw. (20803)

378 ((citizen* or communit* or gender* or public or village?) adj3 (advoca* or input* or ombuds* or represent*)).ti,ab,kw. (31346)

379 Gender Identity/(41227)

380 ((wom#n* or girl* or female? or mother* or wife or wives) adj3 role?).ti,ab,kw. (27434)

381 Social Norms/(10338)

382 ((social* or societ* or cultural* or group?) adj3 (custom* or norm?)).ti,ab,kw. (34970)

383 or/358‐382 (3552165)

384 Nutritional Requirements/(37442)

385 Nutritional Status/(105385)

386 Nutritive Value/(30809)

387 Adolescent Nutritional Physiological Phenomena/(1838)

388 Maternal Nutritional Physiological Phenomena/(13721)

389 Malnutrition/(75398)

390 exp Deficiency Diseases/(260675)

391 (avitaminos#s or hypovitaminos#s or hypo‐vitaminos#s).ti,ab,kw. (8948)

392 ((ascorbic acid? or ferrous ascorbate or hybrin or magnesium ascorbate or magnesium ascorbicum or magnesium di‐L‐ascorbate or magnorbin or sodium ascorbate) adj3 (deficien* or deficit? or inadequa* or insufficien* or intak* or lack* or supplement*)).ti,ab,kw. (4074)

393 scurvy.ti,ab,kw. (4341)

394 ((all‐trans‐retinol or aquasol a or retinol) adj3 (deficien* or deficit? or inadequa* or insufficien* or intak* or lack* or supplement*)).ti,ab,kw. (2037)

395 ((bursine or choline or fagine or vagine) adj3 (deficien* or deficit? or inadequa* or insufficien* or intak* or lack* or supplement*)).ti,ab,kw. (7843)

396 ((folacin or folate or folic acid? or folvite or IFA or pteroylglutamic acid?) adj3 (deficien* or deficit? or inadequa* or insufficien* or intak* or lack*or supplement*)).ti,ab,kw. (17838)

397 (hyperhomocystein?emi* or hyper‐homocystein?emi*).ti,ab,kw. (15508)

398 ((niacin? or enduracin or induracin or lithium nicotinate or nicamin or nico‐400 or nicobid or nicocap or nicolar or nicotinate or nicotinic acid? or wampocap) adj3 (deficien* or deficit? or inadequa* or insufficien* or intak* or lack* or supplement*)).ti,ab,kw. (1808)

399 ((tryptophan or ardeydorm or ardeytropin or L‐tryptophan or levotryptophan or lyphan or naturruhe or optimax or pms‐tryptophan or trofan or tryptacin or tryptan or ratio‐tryptophan) adj3 (deficien* or deficit? or inadequa* or insufficien* or intak* or lack* or supplement*)).ti,ab,kw. (2791)

400 pellagra.ti,ab,kw. (2759)

401 (riboflavin? adj3 (deficien* or deficit? or inadequa* or insufficien* or intak* or lack* or supplement*)).ti,ab,kw. (3214)

402 ((thiamine? or aneurin or thiamin) adj3 (deficien* or deficit? or inadequa* or insufficien* or intak* or lack* or supplement*)).ti,ab,kw. (7904)

403 (pyridoxine? adj3 (deficien* or deficit? or inadequa* or insufficien* or intak* or lack* or supplement*)).ti,ab,kw. (2684)

404 Overnutrition/(5853)

405 exp Obesity/(711110)

406 Growth Disorders/(26038)

407 ((growth adj2 stunt$2) or stunting).ti,ab,kw. (14204)

408 (nutrient? or nutrition* or nutritive or nourish* or malnutrit* or malnourish* or undernutrition or undernourish* or overnutrition or overnourish* or obese or obesity or wasting or underweight or under weight or overweight or over weight).ti,ab,kw. (1787088)

409 Anemia/(242868)

410 exp Anemia, Hypochromic/(46742)

411 (an?emic or an?emia* or chloros#s).ti,ab,kw. (393916)

412 Feeding Behavior/(172106)

413 ((eating or feeding or food or meal? or snack*) adj3 (behavio?r* or habit? or habitual* or pattern* or practice?)).ti,ab,kw. (141188)

414 Diet/(402699)

415 (diet or dieted or diets or dietary or dieting).ti,ab,kw. (1184491)

416 Energy Intake/(93531)

417 ((calori* or energy or protein?) adj3 intak*).ti,ab,kw. (116855)

418 Healthy Diet/(4701)

419 ((eating or feeding or food? or meal? or snack*) adj3 (healthy or unhealthy)).ti,ab,kw. (39049)

420 exp Micronutrients/(688381)

421 (micronutri* or micro‐nutri*).ti,ab,kw. (35918)

422 (trace element? or trace mineral? or micromineral? or micro‐mineral?).ti,ab,kw. (45946)

423 Calcium, Dietary/(31557)

424 Chromium/(59565)

425 Copper/(178475)

426 Iodine/(75382)

427 Iron/(259984)

428 Manganese/(74289)

429 Molybdenum/(21257)

430 Selenium/(58025)

431 Zinc/(170532)

432 (calcium or chromium or copper or fluoride or iodine or iron or manganese or molybdenum or selenium or zinc).ti,ab,kw. (1944286)

433 vitamin*.ti,ab,kw. (520663)

434 exp Dietary Supplements/(89126)

435 ((eating or feeding or food? or meal? or snack*) adj3 supplement*).ti,ab,kw. (25760)

436 n?utr#ceutical?.ti,ab,kw. (14503)

437 Food, Fortified/(11093)

438 ((fortif* or enrich* or functional) adj2 (eating or feeding or food? or meal? or snack*)).ti,ab,kw. (25516)

439 ((home? or school* or work*) adj2 (ate or eaten or eating or eats or breakfast* or lunch* or dinner* or supper* or feed* or food? or meal?)).ti,ab,kw. (19800)

440 Food Supply/(28920)

441 ((eating or feeding or food? or meal? or snack*) adj3 (secure* or securit* or insecure* or insecurit* or supply* or supplie*)).ti,ab,kw. (31461)

442 or/384‐441 (6067800)

443 383 and 442 (295635)

444 Developing Countries/(159704)

445 Africa/or Asia/or Caribbean/or West Indies/or South America/or Latin America/or Central America/(228463)

446 (Afghanistan or Albania or Algeria or Angola or Argentina or Armenia or Armenian or Azerbaijan or Bangladesh or Benin or Byelarus or Byelorussian or Belarus or Belorussian or Belorussia or Belize or Bhutan or Bolivia or Bosnia or Herzegovina or Hercegovina or Botswana or Brazil or Bulgaria or Burkina Faso or Burkina Fasso or Upper Volta or Burundi or Urundi or Cambodia or Khmer Republic or Kampuchea or Cameroon or Cameroons or Cameron or Camerons or Cape Verde or Central African Republic or Chad or China or Colombia or Comoros or Comoro Islands or Comores or Mayotte or Congo or Zaire or Costa Rica or Cote d'Ivoire or Ivory Coast or Cuba or Djibouti or French Somaliland or Dominica or Dominican Republic or East Timor or East Timur or Timor Leste or Ecuador or Egypt or United Arab Republic or El Salvador or Eritrea or Ethiopia or Fiji or Gabon or Gabonese Republic or Gambia or Gaza or Georgia Republic or Georgian Republic or Ghana or Grenada or Guatemala or Guinea or Guiana or Guyana or Haiti or Honduras or India or Maldives or Indonesia or Iran or Iraq or Jamaica or Jordan or Kazakhstan or Kazakh or Kenya or Kiribati or Korea or Kosovo or Kyrgyzstan or Kirghizia or Kyrgyz Republic or Kirghiz or Kirgizstan or Lao PDR or Laos or Lebanon or Lesotho or Basutoland or Liberia or Libya or Macedonia or Madagascar or Malagasy Republic or Malaysia or Malaya or Malay or Sabah or Sarawak or Malawi or Mali or Marshall Islands or Mauritania or Mauritius or Agalega Islands or Mexico or Micronesia or Middle East or Moldova or Moldovia or Moldovian or Mongolia or Montenegro or Morocco or Ifni or Mozambique or Myanmar or Myanma or Burma or Namibia or Nepal or Netherlands Antilles or Nicaragua or Niger or Nigeria or Muscat or Pakistan or Palau or Palestine or Panama or Paraguay or Peru or Philippines or Philipines or Phillipines or Phillippines or Papua New Guinea or Romania or Rumania or Roumania or Rwanda or Ruanda or Saint Lucia or St Lucia or Saint Vincent or St Vincent or Grenadines or Samoa or Samoan Islands or Navigator Island or Navigator Islands or Sao Tome or Senegal or Serbia or Montenegro or Seychelles or Sierra Leone or Sri Lanka or Solomon Islands or Somalia or Sudan or Suriname or Surinam or Swaziland or South Africa or Syria or Tajikistan or Tadzhikistan or Tadjikistan or Tadzhik or Tanzania or Thailand or Togo or Togolese Republic or Tonga or Tunisia or Turkey or Turkmenistan or Turkmen or Uganda or Ukraine or Uzbekistan or Uzbek or Vanuatu or New Hebrides or Venezuela or Vietnam or Viet Nam or West Bank or Yemen or Zambia or Zimbabwe).ti,ab,kw. (2446958)

447 exp africa/or exp africa, northern/or algeria/or egypt/or libya/or morocco/or tunisia/or exp "africa south of the sahara"/or africa, central/or cameroon/or central african republic/or chad/or congo/or "democratic republic of the congo"/or equatorial guinea/or gabon/or africa, eastern/or burundi/or djibouti/or eritrea/or ethiopia/or kenya/or rwanda/or somalia/or south sudan/or sudan/or tanzania/or uganda/or africa, southern/or angola/or botswana/or lesotho/or malawi/or mozambique/or namibia/or south africa/or swaziland/or zambia/or zimbabwe/or africa, western/or benin/or burkina faso/or cape verde/or cote d'ivoire/or gambia/or ghana/or guinea/or guinea‐bissau/or liberia/or mali/or mauritania/or niger/or nigeria/or senegal/or sierra leone/or togo/or americas/or exp caribbean region/or exp west indies/or exp central america/or belize/or costa rica/or el salvador/or guatemala/or honduras/or nicaragua/or panama/or panama canal zone/or latin america/or mexico/or exp south america/or argentina/or bolivia/or brazil/or chile/or colombia/or ecuador/or french guiana/or guyana/or paraguay/or peru/or suriname/or uruguay/or venezuela/or asia/or asia, central/or kazakhstan/or kyrgyzstan/or tajikistan/or turkmenistan/or uzbekistan/or exp asia, southeastern/or borneo/or brunei/or cambodia/or timor‐leste/or indonesia/or laos/or malaysia/or mekong valley/or myanmar/or philippines/or singapore/or thailand/or vietnam/or asia, western/or bangladesh/or bhutan/or india/or sikkim/or middle east/or afghanistan/or bahrain/or iran/or iraq/or israel/or jordan/or kuwait/or lebanon/or oman/or qatar/or saudi arabia/or syria/or turkey/or united arab emirates/or yemen/or nepal/or pakistan/or sri lanka/or far east/or china/or beijing/or macau/or tibet/or korea/or mongolia/or taiwan/or indian ocean islands/or comoros/or madagascar/or mauritius/or reunion/or seychelles/or pacific islands/or exp melanesia/or exp micronesia/or polynesia/or pitcairn island/or exp samoa/or tonga/or prince edward island/or west indies/or "antigua and barbuda"/or bahamas/or barbados/or cuba/or dominica/or dominican republic/or grenada/or guadeloupe/or haiti/or jamaica/or martinique/or netherlands antilles/or puerto rico/or "saint kitts and nevis"/or saint lucia/or "saint vincent and the grenadines"/or "trinidad and tobago"/or united states virgin islands/or oceania/(2424512)

448 ((developing or less* developed or under developed or underdeveloped or middle income or low* income or underserved or under served or deprived or poor*) adj (countr* or nation? or population? or region? or world or state*)).ti,ab,kw. (234706)

449 ((developing or less* developed or under developed or underdeveloped or middle income or low* income or poor*) adj econom*).ti,ab,kw. (2988)

450 (low* adj (gdp or gnp or gross domestic or gross national)).ti,ab,kw. (643)

451 (low adj3 middle adj3 countr*).ti,ab,kw. (30878)

452 (lmic or lmics or third world or lami countr*).ti,ab,kw. (15985)

453 transitional countr*.ti,ab,kw. (445)

454 or/444‐453 (3687965)

455 443 and 454 (30919)

456 Women/(8405706)

457 Pregnant Women/(69649)

458 Female/and (Adolescent/or Adult/or Young Adult/) (9600899)

459 (woman* or women* or matern* or mother* or wife or wives or pregnan*).ti,ab,kw. (4324318)

460 ((female? or girl*) adj5 (adolescen* or adult* or child‐bearing or childbearing or highschool* or school* or teen* or marry* or marriage* or marrie? or menstru* or post‐pubescen* or postpubescen* or post‐puberty or postpuberty or reproductive or spous* or youth* or young adult?)).ti,ab,kw. (284320)

461 ((female? or girl or girls) adj2 (ten year? or ten yr? or 10 year? or 10 yr? or eleven year? or eleven yr? or 11 year? or 11 yr? or twelve year? or twelve yr? or 12 year? or 12 yr?)).ti,ab,kw. (28338)

462 ((female? or girl or girls) adj2 (age? or old) adj3 ("10" or ten or "11" or eleven or "12" or twelve)).ti,ab,kw. (65402)

463 young girl?.ti,ab,kw. (9994)

464 or/456‐463 (15074516)

465 455 and 464 (18238)

466 465 use coch,cctr,dare,clhta,cleed [COCHRANE RECORDS] (925)

467 114 or 244 or 357 or 466 [ALL DATABASES] (18543)

468 limit 467 to yr = "2016‐current" [Limit not valid in DARE; records were retained] (5061)

469 remove duplicates from 468 (3740)

470 limit 467 to yr = "2011‐2015" [Limit not valid in DARE; records were retained] (5957)

471 remove duplicates from 470 (4296)

472 limit 467 to yr = "2000‐2010" [Limit not valid in DARE; records were retained] (4760)

473 remove duplicates from 472 (3258)

474 467 not (468 or 470 or 472) (2767)

475 remove duplicates from 474 (2200)

476 469 or 471 or 473 or 475 [TOTAL UNIQUE RECORDS] (13492)

477 476 use medall [MEDLINE UNIQUE RECORDS] (6468)

478 476 use emczd [EMBASE UNIQUE RECORDS] (5659)

479 476 use medal, emczd, coch, cctr, dare, cleed, clhta (12605)

480 476 not 479 [PSYCINFO UNIQUE RECORDS] (887)

481 476 use coch [DSR UNIQUE RECORDS] (4)

482 476 use dare [DARE UNIQUE RECORDS] (1)

483 476 use cleed [NHS UNIQUE RECORDS] (0)

484 476 use clhta [HTA UNIQUE RECORDS] (0)

485 476 use CCTR [CENTRAL UNIQUE RECORDS] (473)

## APPENDIX B DATA EXTRACTION TEMPLATE

### Quantitative Study Codebook

Where possible, provide direct quotes and page numbers to support your extractions.

**Table:**

Code	Description
Study ID	from Covidence
Date coded	
Coder initials	
Correspondence required	Add a note here if authors will need to be contacted to request additional information
Reference identification	
Title	
First author	
Year	
Type of publication	(drop‐down) Journal article/Book/Conference abstract/Dissertation/Report/Other
Other	Define “other” publication type
Funder	
Country(ies)	
City(ies) and region(s) (if applicable)	
Start date of study	
End date of study	
Study duration	
Aim of the study	
Authors’ definition of empowerment	
Authors’ rationale for empowerment approach	
Methods	
Study design	(drop‐down) RCT/cRCT/CBA/ITS/cITS/DID/RDD/PSM/other
Other	Define “other” study design type
Study design description	
Statistical method	Describe method used and comment on appropriateness.
Intervention	
Number of treatment arms	(drop‐down) 1–10
Description of treatment arm 1	Describe the treatment or intervention group as outlined in the study
Description of treatment arm 2 (continue adding for multi‐arm interventions as needed)	
Comparison group	Describe the nature of the comparison group
Type of nutrition intervention	(drop‐down—select all that apply) Micronutrient supplementation/Fortification/Education or Counselling/School feeding/Other supplemental nutrition/Other
Description of nutrition intervention	
Type of agency intervention	(drop‐down—select all that apply) Mentorship program/Leadership skills training/Technical or occupational skills training/Program leadership role for girls/Peer education/Peer support group/Other
Description of agency intervention	
Type of opportunity structure intervention	(drop‐down—select all that apply) Cash transfer program/Savings and loan program/Education support/Girls’ rights advocacy or education (with parents, teachers, community influencers)/Prevention of early marriage and pregnancy/Other
Description of opportunity structure intervention	
Intervention setting	(drop‐down—select all that apply) School/Community/Home/Health Facility/Other
Other	Describe “other” intervention setting
Geography	(drop‐down—select all that apply) Urban/Rural/Remote/Slum/Other
Other	Describe other geographic setting
Intervention administrator	(drop‐down) Foreign government/local or national government/nongovernment organization/community‐based organization/Academic institution/Other
Other	Describe other intervention administrator
Intervention provider	(drop‐down—select all that apply) Community health workers/Health facility staff/Teachers/Peers/Parents/Volunteers/Other
Other	Describe other intervention provider
Provider training	Describe the training of providers to deliver intervention
Recruitment	Describe procedures for recruiting participants
Attrition rate	The proportion of participants lost during the intervention or during follow‐up.
Reach	Describe the degree to which the intended audience participated in an intervention by “their presence”
Dose	Describe the proportion or amount of an intervention delivered to participants. For example, measures of frequency, duration, and/or intensity
Fidelity/integrity	Describe the degree to which the intervention was delivered as intended.
Adaptation	Describe the degree to which the program content was intentionally changed during implementation to improve programme effectiveness
Prior needs assessment	Describe any needs assessment that was conducted to inform intervention design
Reminders	Describe any prompts or reminders sent to participants to attend/participate in intervention
Quality	Describe any findings related to the quality of quality of intervention materials/resources (e.g., curriculum, training, and policy)
Cultural appropriateness	Describe any findings related to the cultural appropriateness of the intervention
Contamination	Describe any unintentional delivery of intervention to the control group or inadvertent failure to deliver intervention to experimental group
Cointervention	Describe any instances where interventions other than the treatment were applied differently to intervention conditions
Participant engagement	Describe participant's interaction with or receptivity to the intervention (i.?e., what they think or how they feel about the intervention)
Contextual factors	Describe any social, built, and political factors internal (e.g., partnerships) and external to the intervention environment (e.g., social norms) that shape implementation.
Study population	
Number of participants	
Number in intervention group 1	
Number in intervention group 2 (continue adding for each treatment arm as needed)	
Number in comparison group	
Age range of study participants	Enter age range of study target population, for example, 15–49 years
Age subgroups	List all age subgroups for which data are provided, for example, study covers 15‐49 years, and analyses were done separately for 15–19, 20–24, 25–49 years
Did the study population also include men or boys?	(drop‐down) Yes/No/Unclear
Race/ethnicity/language	Describe
Religion	Describe
Socioeconomic status	Describe
Disability, physical health	Describe
Includes pregnant participants	(drop‐down) Yes/No/Unclear
Includes young mothers	(drop‐down) Yes/No/Unclear
Conflict setting	(drop‐down) Yes/No/Unclear
Food insecure setting	(drop‐down) Yes/No/Unclear
Outcomes	
Primary outcomes	Define outcomes and how they are measured
Secondary outcomes	Define outcomes and how they are measured
Method of assessing outcomes	Describe methods for assessing outcome measures
Validity and reliability of outcomes measures	Describe primary authors’ views on the validity and reliability of outcome measures
Follow‐up of nonrespondents	Describe the methods used to follow‐up nonrespondents
Timing of outcome assessment	Describe frequency of assessment, length of follow‐up, and so forth
Results	
Number of participants in Treatment Arm 1	
Number of participants in Treatment Arm 2 (continue adding for each treatment arm as needed)	
Number of participants in comparison group	
*For each outcome (and each intervention vs comparison)…*	
Direction of effect	(drop‐down) Effect favours treatment/Effect favours comparison/Effect favours neither/Cannot tell
Effect statistically significant?	(drop‐down) Yes/No/Cannot tell
Treatment sample size	
Control/comparison sample size	
Number of missing participants	
Nature of the measure	(drop‐down) Continuous/Dichotomous/Other
Nature of the measure if "other" selected	
*If outcome measure is continuous…*	
Comparison group mean (select type of measure reported)	(drop‐down) endline, change (endline‐baseline), change (baseline‐endline)
Comparison group mean (enter value)	
Treatment group mean (select type of measure if reported)	(drop‐down) endline, change (endline‐baseline), change (baseline‐endline), NA
Treatment group mean (enter value or values if multi‐arm)	
Effect estimate (select type of measure if reported)	(drop‐down) mean difference, standardized mean difference, mean ratio, NA
Effect estimate (enter value or values if multi‐arm)	
Is reported effect estimate adjusted?	(drop‐down) Yes/No/Cannot tell
Comparison group standard deviation	
Comparison group standard error	
Treatment group(s) standard deviation(s)	
Treatment group(s) standard error(s)	
Effect estimate standard error(s)	
Confidence interval(s)	
Test‐statistic value(s) and/or *p* value(s)	
Adjusted effect estimate standard error(s)	
Confidence interval(s) of adjusted effect estimate(s)	
Test statistic value(s) and/or *p* value(s) of adjusted effect estimate(s)	
*If outcome measure is dichotomous…*	
Treatment group number of participants who experienced a change (in each intervention group if multi‐arm)	
Comparison group number of participants who experienced a change	
Treatment group proportion of participants who experienced a change (in each intervention group if multi‐arm)	
Comparison group proportion of participants who experienced a change	
Effect estimate (select type reported)	(drop‐down) risk difference (difference in proportions), relative risk (ratio of the proportions), odds ratio (ratio of the odds), NA
Effect estimate(s) (enter value(s))	
Is reported effect estimate adjusted?	(drop‐down) Yes/No/Unclear
Effect estimate standard error(s)	
Confidence interval(s)	
Test‐statistic value(s) and/or *p* value(s)	
Adjusted effect estimate standard error(s)	
Confidence interval(s) of adjusted effect estimate(s)	
Test statistic value(s) and/or *p* value(s) of adjusted effect estimate(s)	
*Adverse events*	
Adverse events	List all adverse events of integrating women's empowerment, as described by primary authors.

### Qualitative study codebook

Where possible, provide direct quotes and page numbers to support your extractions.

**Table:**

Code	Description
Study ID	from Covidence
Date coded	
Coder initials	
Correspondence required	Add a note here if authors will need to be contacted to request additional information
Reference identification	
Title	
First author	
Year	
Type of publication	(drop‐down) Journal article/Book/Conference abstract/Dissertation/Report/Other
Other	Define “other” publication type
Funder	
Country	
City and region (if applicable)	
Start date of study	
End date of study	
Aim of the study	
Authors’ definition of empowerment	
Authors’ rationale for empowerment approach	
Methods	
Study design description	
Study duration	
Participant inclusion/exclusion criteria	
*Quality assessment (CASP)*	
Was there a clear statement of the aims of the research?	(drop‐down) Yes/No/Unclear
Support for judgement (incl. pg #)	
Is a qualitative methodology appropriate?	(drop‐down) Yes/No/Unclear
Support for judgement (incl. pg #)	
Was the research design appropriate to address the aims of the research?	(drop‐down) Yes/No/Unclear
Support for judgement (incl. pg #)	
Was the recruitment strategy appropriate to the aims of the research?	(drop‐down) Yes/No/Unclear
Support for judgement (incl. pg #)	
Was the data collected in a way that addressed the research issue?	(drop‐down) Yes/No/Unclear
Support for judgement (incl. pg #)	
Has the relationship between researcher and participants been adequately considered?	(drop‐down) Yes/No/Unclear
Support for judgement (incl. pg #)	
Have ethical issues been taken into consideration?	(drop‐down) Yes/No/Unclear
Support for judgement (incl. pg #)	
Was the data analysis sufficiently rigorous?	(drop‐down) Yes/No/Unclear
Support for judgement (incl. pg #)	
Is there a clear statement of findings?	(drop‐down) Yes/No/Unclear
Support for judgement (incl. pg #)	
How valuable is the research?	(drop‐down) Yes/No/Unclear
Support for judgement (incl. pg #)	
Study population	
Number of participants	
Study scale	Small (e.g., one/several village)/large (e.g., district, region, province)
Describe scale	
Study setting	(drop‐down) Urban/Rural/Informal‐urban/Remote/Other
Describe setting	
Target	(drop‐down) Individual/Household/Community/Other
Describe target	
Age range of study participants	Enter age range of study target population, for example, 15‐49 years
Age subgroups	List all female age subgroups for which data are provided, for example, study covers 15‐49 years, and analyses were done separately for 15‐19 years, 20‐24 years, 25‐49 years
Did the study population also include men or boys?	(drop‐down) Yes/No/Unclear
Race/ethnicity/language	Describe
Religion	Describe
Socioeconomic status	Describe
Disability, physical health	Describe
Includes pregnant participants	(drop‐down) Yes/No/Unclear
Includes young mothers	(drop‐down) Yes/No/Unclear
Conflict setting	(drop‐down) Yes/No/Unclear
Food insecure setting	(drop‐down) Yes/No/Unclear
Intervention	
Type of nutrition intervention	(drop‐down—select all that apply) Micronutrient supplementation/Fortification/Education or Counselling/School feeding/Other supplemental nutrition/Other
Description of nutrition intervention	
Type of agency intervention	(drop‐down—select all that apply) Mentorship program/Leadership skills training/Technical or occupational skills training/Program leadership role for girls/Peer education/Peer support group/Other
Description of agency intervention	
Type of opportunity structure intervention	(drop‐down—select all that apply) Cash transfer program/Savings and loan program/Education support/Girls’ rights advocacy or education (with parents, teachers, community influencers)/Prevention of early marriage and pregnancy/Other
Description of opportunity structure intervention	
Intervention setting	(drop‐down—select all that apply) School/Community/Home/Health Facility/Other
Describe intervention setting	
Intervention administrator	(drop‐down) Foreign government/local or national government/nongovernment organization/community‐based organization/Academic institution/Other
Describe intervention administrator	
Intervention provider	(drop‐down—select all that apply) Community health workers/Health facility staff/Teachers/Peers/Parents/Volunteers/Other
Describe intervention provider	
Provider training	Describe the training of providers to deliver intervention
Recruitment	Describe procedures for recruiting participants
Attrition rate	The proportion of participants lost during the intervention or during follow‐up.
Reach	Describe the degree to which the intended audience participated in an intervention by “their presence”
Dose	Describe the proportion or amount of an intervention delivered to participants. For example, measures of frequency, duration, and/or intensity
Fidelity/integrity	Describe the degree to which the intervention was delivered as intended.
Adaptation	Describe the degree to which the program content was intentionally changed during implementation to improve programme effectiveness
Prior needs assessment	Describe any needs assessment that was conducted to inform intervention design
Reminders	Describe any prompts or reminders sent to participants to attend/participate in intervention
Quality	Describe any findings related to the quality of quality of intervention materials/resources (e.g., curriculum, training, and policy)
Cultural appropriateness	Describe any findings related to the cultural appropriateness of the intervention
Contamination	Describe any unintentional delivery of intervention to the control group or inadvertent failure to deliver intervention to experimental group
Cointervention	Describe any instances where interventions other than the treatment were applied differently to intervention conditions
Participant engagement	Describe participant's interaction with or receptivity to the intervention (i.?e., what they think or how they feel about the intervention)
Contextual factors	Describe any social, built, and political factors internal (e.g., partnerships) and external to the intervention environment (e.g., social norms) that shape implementation.
Measures	
*Feasibility (the extent to which an intervention is practical or viable in a particular context or situation)*	
Cultural sensitivity of program	
Participant engagement	
Community/public commitment	
*Appropriateness (the extent to which an intervention or activity fits with a particular context or situation)*	
Participants’ views on programme acceptability	
Implementers’ views on programme acceptability	
Community support for programme	
*Meaningfulness (the extent to which an intervention or activity is positively experienced by an individual or group)*	
Participants’ views on programme as a positive or negative experience	
Participants’ views on benefits and costs of participation	
Participants’ awareness of own nutritional needs	
Participants’ motivation to act for improved nutrition	
Community desire to support participants to act for improved nutrition	
*Adverse events*	
Adverse events	List all adverse events of integrating women's empowerment, as described by primary authors.
